# Identification of New Virulence Factors and Vaccine Candidates for *Yersinia pestis*

**DOI:** 10.3389/fcimb.2017.00448

**Published:** 2017-10-17

**Authors:** Jourdan A. Andersson, Jian Sha, Tatiana E. Erova, Eric C. Fitts, Duraisamy Ponnusamy, Elena V. Kozlova, Michelle L. Kirtley, Ashok K. Chopra

**Affiliations:** ^1^Institute for Translational Sciences, University of Texas Medical Branch, Galveston, TX, United States; ^2^Department of Microbiology and Immunology, University of Texas Medical Branch, Galveston, TX, United States; ^3^Institute for Human Infections and Immunity, University of Texas Medical Branch, Galveston, TX, United States; ^4^WHO Collaborating Center for Vaccine Development, University of Texas Medical Branch, Galveston, TX, United States; ^5^Center for Biodefense and Emerging Infectious Diseases, University of Texas Medical Branch, Galveston, TX, United States

**Keywords:** *Yersinia pestis*, mouse models of bubonic and pneumonic plague, type 6 secretion system and effectors, phagocytosis, intracellular survival

## Abstract

Earlier, we reported the identification of new virulence factors/mechanisms of *Yersinia pestis* using an *in vivo* signature-tagged mutagenesis (STM) screening approach. From this screen, the role of *rbsA*, which encodes an ATP-binding protein of ribose transport system, and *vasK*, an essential component of the type VI secretion system (T6SS), were evaluated in mouse models of plague and confirmed to be important during *Y. pestis* infection. However, many of the identified genes from the screen remained uncharacterized. In this study, in-frame deletion mutants of *ypo0815, ypo2884, ypo3614-3168 (cyoABCDE)*, and *ypo1119-1120*, identified from the STM screen, were generated. While *ypo0815* codes for a general secretion pathway protein E (GspE) of the T2SS, the *ypo2884*-encoded protein has homology to the βγ crystallin superfamily, *cyoABCDE* codes for the cytochrome o oxidase operon, and the *ypo1119-1120* genes are within the Tol-Pal system which has multiple functions. Additionally, as our STM screen identified three T6SS-associated genes, and, based on *in silico* analysis, six T6SS clusters and multiple homologs of the T6SS effector hemolysin-coregulated protein (Hcp) exist in *Y. pestis* CO92, we also targeted these T6SS clusters and effectors for generating deletion mutants. These deletion mutant strains exhibited varying levels of attenuation (up to 100%), in bubonic or pneumonic murine infection models. The attenuation could be further augmented by generation of combinatorial deletion mutants, namely Δ*lpp*Δ*ypo0815*, Δ*lpp*Δ*ypo2884*, Δ*lpp*Δ*cyoABCDE*, Δ*vasK*Δ*hcp6*, and Δ*ypo2720-2733*Δ*hcp3*. We earlier showed that deletion of the *lpp* gene, which encodes Braun lipoprotein (Lpp) and activates Toll-like receptor-2, reduced virulence of *Y. pestis* CO92 in murine models of bubonic and pneumonic plague. The surviving mice infected with Δ*lpp*Δ*cyoABCDE*, Δ*vasK*Δ*hcp6*, and Δ*ypo2720-2733*Δ*hcp3* mutant strains were 55–100% protected upon subsequent re-challenge with wild-type CO92 in a pneumonic model. Further, evaluation of the attenuated T6SS mutant strains *in vitro* revealed significant alterations in phagocytosis, intracellular survival in murine macrophages, and their ability to induce cytotoxic effects on macrophages. The results reported here provide further evidence of the utility of the STM screening approach for the identification of novel virulence factors and to possibly target such genes for the development of novel live-attenuated vaccine candidates for plague.

## Introduction

*Yersinia pestis*, a Gram-negative, facultative anaerobic bacterium, is classified as a Tier-1 select agent by the Centers for Disease Control and Prevention (CDC), and a re-emerging human pathogen by the World Health Organization (WHO) as it remains a prevalent, global public health threat (Perry and Fetherston, 1997; Prentice and Rahalison, 2007). *Y. pestis* leads to three disease manifestations in humans, namely bubonic, septicemic, and pneumonic plague. Unfortunately, there is no Food and Drug Administration (FDA)-approved vaccine against plague that is available and the only treatment option for *Y. pestis*-inflicted patients is antibiotic therapy (Russell et al., 1998; Inglesby et al., 2000; Peterson et al., 2010). Isolation of multi-antibiotic resistant strains from patients with plague (Galimand et al., 1997; Guiyoule et al., 2001) and engineering of antibiotic-resistant strains of *Y. pestis* by some countries (Inglesby et al., 2000; Ligon, 2006) to be used as a biological warfare agent highlight the need for not only a better understanding of the pathogenesis of *Y. pestis* infections, but also for the development of an effective vaccine to combat this deadly pathogen.

Recombinant subunit plague vaccines comprised of capsular antigen F1 and the type 3 secretion system (T3SS) component and effector low-calcium response V antigen (LcrV), are currently in clinical trials and have shown promise in rodent models (Rosenzweig et al., 2011; Feodorova and Motin, 2012). However, protection is inconsistent in different species of non-human primates (e.g., Cynomolgus macaques vs. African green monkeys) with antibody titers not correlating with protection (Smiley, 2008; Williamson et al., 2011). Further, the F1 and LcrV antibody titers generated by the subunit vaccine(s) in humans vary significantly and the F1-LcrV-based vaccines generate a poor T cell response (Williamson et al., 2005). Recent studies, including ours, have indicated that cell mediated immune responses are crucial in providing protection against *Y. pestis* infections, specifically pneumonic plague, and, as such, subunit vaccines are not likely to be highly effective as they primarily generate an antibody-mediated immune response (Parent et al., 2005; Lin et al., 2011; Van Lier et al., 2014; Tiner et al., 2015b). Furthermore, such vaccines will be relatively ineffective against infections caused by F1-minus strains of *Y. pestis*, which exist in nature and are equally virulent, or those harboring highly diverged variants of LcrV (Anisimov et al., 2010; Sha et al., 2011). Thus, live-attenuated vaccines offer a substantial advantage in triggering both protective humoral and cell-mediated immune responses. Indeed, a live-attenuated vaccine strain, *Y. pestis* EV76, which lacks the pigmentation locus required for iron acquisition, provides protection against both bubonic and pneumonic plague in humans and is used in endemic regions of China and the former states of the Soviet Union (Smiley, 2008; Williamson, 2009). However, this strain is highly reactogenic and can induce disease similar to that of wild-type (WT) bacteria in individuals with underlying diseases, such as hemochromatosis [Centers for Disease Control and Prevention (CDC), 2011]. Consequently, there is an urgent need for the development of alternative vaccine candidates, particularly live-attenuated ones, as they would trigger both arms of the immune responses in the host.

Recently, we utilized a signature-tagged mutagenesis (STM) approach to identify potential novel virulence factors of *Y. pestis* for the development of rationally designed live-attenuated vaccine candidate strains (Ponnusamy et al., 2015). Using this screening technique, 15 transposon mutants were identified to be attenuated in either a mouse model of bubonic plague or a pneumonic mouse model (Ponnusamy et al., 2015). The pathogenic roles of *rbsA* (*ypo2500*), a gene encoding a putative sugar transport ATP-binding protein; *vasK* (*ypo3603*), a component of the type VI secretion system (T6SS) cluster G (Table 1); and *ypo0498*, a gene within a putative T6SS cluster A (Table 1), were further studied by generating in-frame deletion mutants. *In vivo* analysis of the single Δ*vasK* and the double deletion mutant Δ*lpp*Δ*vasK*, with the *lpp* gene encoding Braun lipoprotein which activates host's Toll-like receptor 2 (TLR-2) signaling (Sha et al., 2008), we reported for the first time the involvement of the T6SS in *Y. pestis* virulence (Ponnusamy et al., 2015). As *ypo0498* is part of a T6SS cluster A and its in-frame deletion did not affect virulence *in vivo*, this is indicative that not all T6SS clusters of *Y. pestis* are equally functional.

**Table 1:** Putative T6SS loci, Hcp proteins, and PAAR motif-containing proteins identified in the *Y. pestis* genome.

**Cluster name and location**	**Putative Hcp proteins (% identity to Hcp of *A. dhakensis*)**	**PAAR motif-containing proteins and their biologically-active domains**
A: *ypo0498-ypo0518*	Hcp1: YPO0973 (31%); part of Cluster B	YPO0762 tRNA nuclease (WapA)
B: *ypo0966-ypo0984*	Hcp2: YPO1470 (31%);part of Cluster C	YPO0866
C: *ypo1458-ypo1493*	Hcp3: YPO2793 (32%)	YPO0873 pyocin S type
E: *ypo2715-ypo2733*	Hcp4: YPO2868 (34%)	YPO1484 Toxin 60 (RNase toxin)
F: *ypo2927-ypo2954*	Hcp5: YPO2962 (32%)	YPO3615 tRNA nuclease (WapA)
G: *ypo3588-ypo3615*	Hcp6: YPO3708 (82%)	

Interestingly, a total of three (*vasK, ypo498*, and *ypo1484*; part of this study) T6SS component or effector-encoding genes were identified from our STM screen (Ponnusamy et al., 2015). The T6SS is highly conserved amongst Gram-negative bacteria and is involved in the secretion and delivery of effector proteins to both prokaryotic and eukaryotic cells (Filloux, 2013). The T6SS plays a vital role in the virulence of several pathogens including *Burkholderia mallei, B. pseudomallei, Francisella tularensis, Vibrio cholerae, Pseudomonas aeruginosa, Salmonella enterica* serovar Typhimurium, and *Aeromonas dhakensis* (previously classified as *Aeromonas hydrophila*; Parsons and Heffron, 2005; Mougous et al., 2006; Pukatzki et al., 2006; Schell et al., 2007; Suarez et al., 2008; Burtnick et al., 2011; Sha et al., 2013b; Grim et al., 2014; Rigard et al., 2016). For *Y. pestis, in silico* analysis of the genome revealed six potential T6SS clusters (A–C, E–G), six potential hemolysin co-regulated protein (Hcp, designated from 1 to 6)-encoding effector genes, and five potential proline-alanine-alanine-arginine (PAAR) repeat-containing effector-encoding genes (Table 1); however, their roles in *Y. pestis* virulence remain largely unknown.

To further study the pathogenic roles of T6SSs and their effectors, we generated mutants individually deleted for five T6SS clusters (B, C, and E–G), four *hcp* gene homologs (*hcp3-6)*, and genes for three PAAR motif repeat-containing protein (Table S1). One of the latter genes, *ypo1484*, was identified during our STM screen (Ponnusamy et al., 2015). The T6SS Cluster A (*ypo0498-ypo0518*) (Table 1) was previously characterized by us and shown to play no role in *Y. pestis* virulence in either bubonic or pneumonic murine plague models, although attenuation was observed in an *in vitro* assay when the intracellular survival of the mutant was evaluated in J774.1 murine macrophages (Robinson et al., 2009).

In addition to T6SS-associated genes, we examined the contribution of other genes identified from our STM screen in inducing plague by generating in-frame deletion mutants. These genes included *ypo0815*, encoding a general secretion pathway protein E (GspE) of the T2SS, *ypo2884*, which encodes a protein with homology to the βγ crystallin superfamily, and *ypo3164*, encoding the cytochrome *o* ubiquinol oxidase subunit II, a component of the cytochrome o oxidase *(cyoABCDE)* operon. In addition to these three genes, an intergenic region between the *ypo1119* and *ypo1120* genes was also identified through our STM screen (Ponnusamy et al., 2015). The *ypo1119-1120* genes are within the Tol-Pal system, which has multiple functions including maintaining bacterial membrane integrity (Lazzaroni et al., 1999). These various deletion mutants exhibited varying levels of attenuation in murine models of bubonic or pneumonic plague in comparison to WT *Y. pestis* CO92. Furthermore, attenuation could be augmented through the generation of combinatorial deletion mutants as observed with Δ*lpp*Δ*ypo0815*, Δ*lpp*Δ*ypo2884*, Δ*lpp*Δ*cyoABCDE*, Δ*vasK*Δ*hcp6*, and Δ*ypo2720-2733*Δ*hcp3*. We chose to generate combinatorial mutants inclusive of the *lpp* gene as the splenocytes and macrophages of mice infected with the Δ*lpp* mutant of *Y. pestis* KIM/D27 strain had higher levels of interferon (IFN)-γ, interleukin (IL)-2, and IL-12 p40 compared to the levels in cells infected with the parental bacteria (Liu et al., 2010). Increased production of IL-12 signified that the Δ*lpp* mutant would not be able to survive well within host cells (Liu et al., 2010). Further, we showed that Lpp was required for CO92 to survive intracellularly in macrophages, an effect modulated by the global stress response protein, GsrA (Galindo et al., 2010). Mice that survived intranasal (i.n.) challenge, to model pneumonic plague infection, with the double deletion mutants Δ*lpp*Δ*cyoABCDE*, Δ*vasK*Δ*hcp6*, and Δ*ypo2720-2733*Δ*hcp3*, were also observed to be significantly protected from subsequent re-challenge with WT CO92. As the two T6SS double deletion mutants, Δ*vasK*Δ*hcp6* and Δ*ypo2720-2733*Δ*hcp3*, exhibited the highest levels of protection against subsequent pneumonic re-challenge, *in vitro* studies were performed to further elucidate the mechanisms of attenuation for the generated T6SS deletion mutants. We found that the attenuated mutant strains exhibited distinct phenotypes in terms of induction of host cell cytotoxicity, phagocytosis by murine macrophages, and intracellular survival in such macrophages. These results indicated that the T6SS effectors and clusters have distinct roles in *Y. pestis* virulence. Our data also provided further evidence of the utility of the STM screening approach for the identification of novel virulence factors to be targeted for deletion and rational design of potential new generation live-attenuated vaccine candidate(s).

## Materials and methods

### Bacterial strains and cell culture

The bacterial strains used in this study are described in Table S1. *Y. pestis* strains were grown in heart infusion broth (HIB) (Difco; Voigt Global Distribution, Inc., Lawrence, KS) at 28 or 37°C with constant shaking at 180 rpm, or grown for 48 h on 5% sheep blood agar (SBA) (Teknova, Hollister, CA) or HIB agar plates. As appropriate, the organisms were cultivated in the presence of antibiotics ampicillin and kanamycin at concentrations of 100 and 50 μg/ml, respectively. All experiments with *Y. pestis* strains were performed in the CDC-approved select agent laboratory within the Galveston National Laboratory (GNL), University of Texas Medical Branch (UTMB).

The RAW 264.7 murine macrophage cell line (American Type Culture Collection, [ATCC], Manassas, VA) was maintained in Dulbecco's modified eagle medium (DMEM) with 10% fetal bovine serum supplemented with 1% L-glutamine (Cellgro, Manassas, VA) and 1% penicillin-streptomycin (Invitrogen, Carlsbad, CA) at 37°C with 5% CO_2_.

### Construction of single gene, T6SS cluster, and combinatorial deletion mutants of *Y. pestis* CO92

To construct in-frame single gene deletion and cluster deletion mutants of CO92, the λ phage recombination system was used as previously described (Datsenko and Wanner, 2000; Ponnusamy et al., 2015). Briefly, the parent strains were transformed with plasmid pKD46 (Table S1) and grown in the presence of 1 mM l-arabinose to induce the expression of the λ phage recombinase gene on pKD46. The parent strains were processed for the preparation of electroporation-competent cells (Ponnusamy et al., 2015). The electrocompetent cells were then transformed with 0.5–1.0 μg of the linear ds DNA constructs carrying the kanamycin resistance (Km^r^) gene cassette, which was immediately flanked by the bacterial flippase recognition target (FRT) sequence, followed on either side by 50 bp of DNA sequences homologous to the 5′ and 3′ ends of the gene to be deleted from the parent strains. Plasmid pKD46 was cured from the mutants that had successful Km^r^ gene cassette integration at the correct location by growing the bacteria at 37°C. The latter mutants were transformed with plasmid pEF01 (Table S1) (Fitts et al., 2016) to excise the Km^r^ gene cassette. Plasmid pEF01 was then cured from the Km^s^ clones by growing them at 37°C, followed by selection in a medium containing 5% sucrose. To confirm the in-frame deletion, mutants showing sensitivity to kanamycin and ampicillin were tested by polymerase chain reaction (PCR) using appropriate primer pairs (Table S2) and sequencing of the PCR products.

### Testing attenuation of the *Y. pestis* CO92 mutant strains in mouse models of bubonic and pneumonic plague

**Figure 1:**
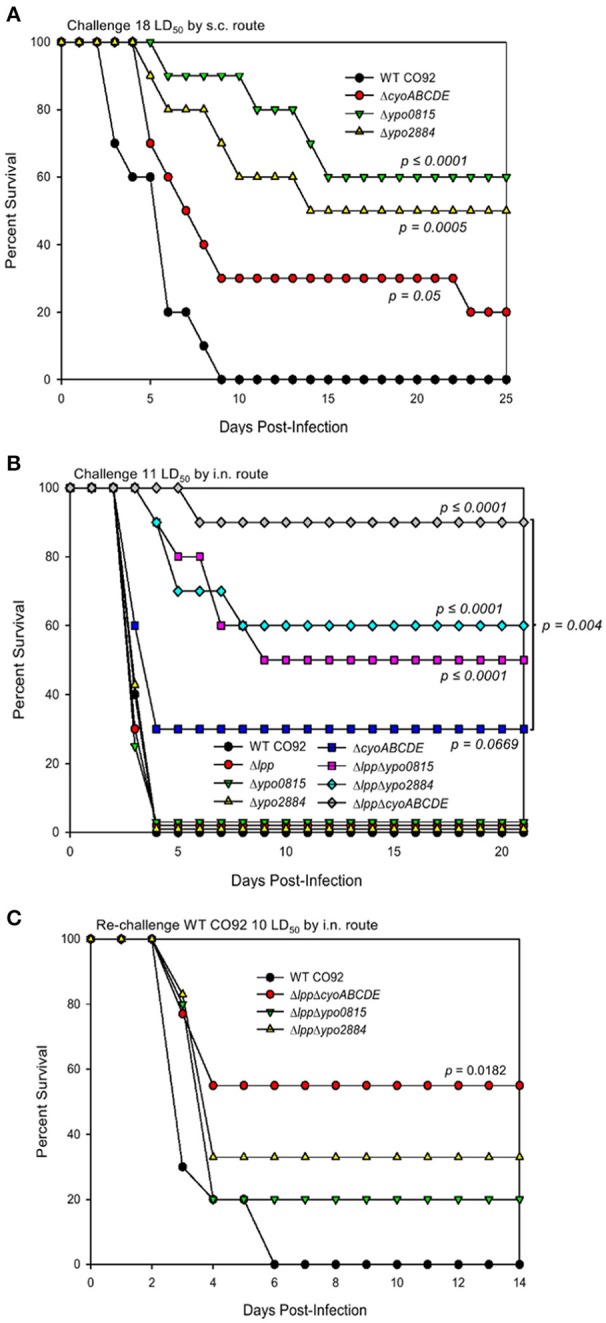
Role of *Y. pestis* CO92 genes identified through the signature-tagged mutagenesis screen in virulence using bubonic and pneumonic plague mouse models. Survival analysis of female Swiss-Webster mice infected with WT CO92 or one of the indicated mutant strains (*n* = 10) in a bubonic plague model at the dosage equivalent to 18 LD_50_ of WT *Y. pestis* CO92 **(A)** or in a pneumonic model at the dosage equivalent to 11 LD_50_ of WT *Y. pestis* CO92 **(B)**. Animals were monitored for up to 25 days p.i. **(C)** Survived mice (*n* = 5–9) following the initial i.n. mutant infections **(B)** and a group of naïve control mice (*n* = 5) were subsequently challenged with 10 LD_50_ WT CO92 and observed for morbidity/mortality for 14 days p.i. (35 days total from initial infection). The data were analyzed for significance by using Kaplan–Meier survival estimates. The *P*-values were determined based on comparison of animal survival for each mutant to the survival of WT CO92-infected control animals or the groups indicated by a line. Two independent experiments were performed and the data from the second set constituting a group size of 10 animals/group were presented.

**Figure 2:**
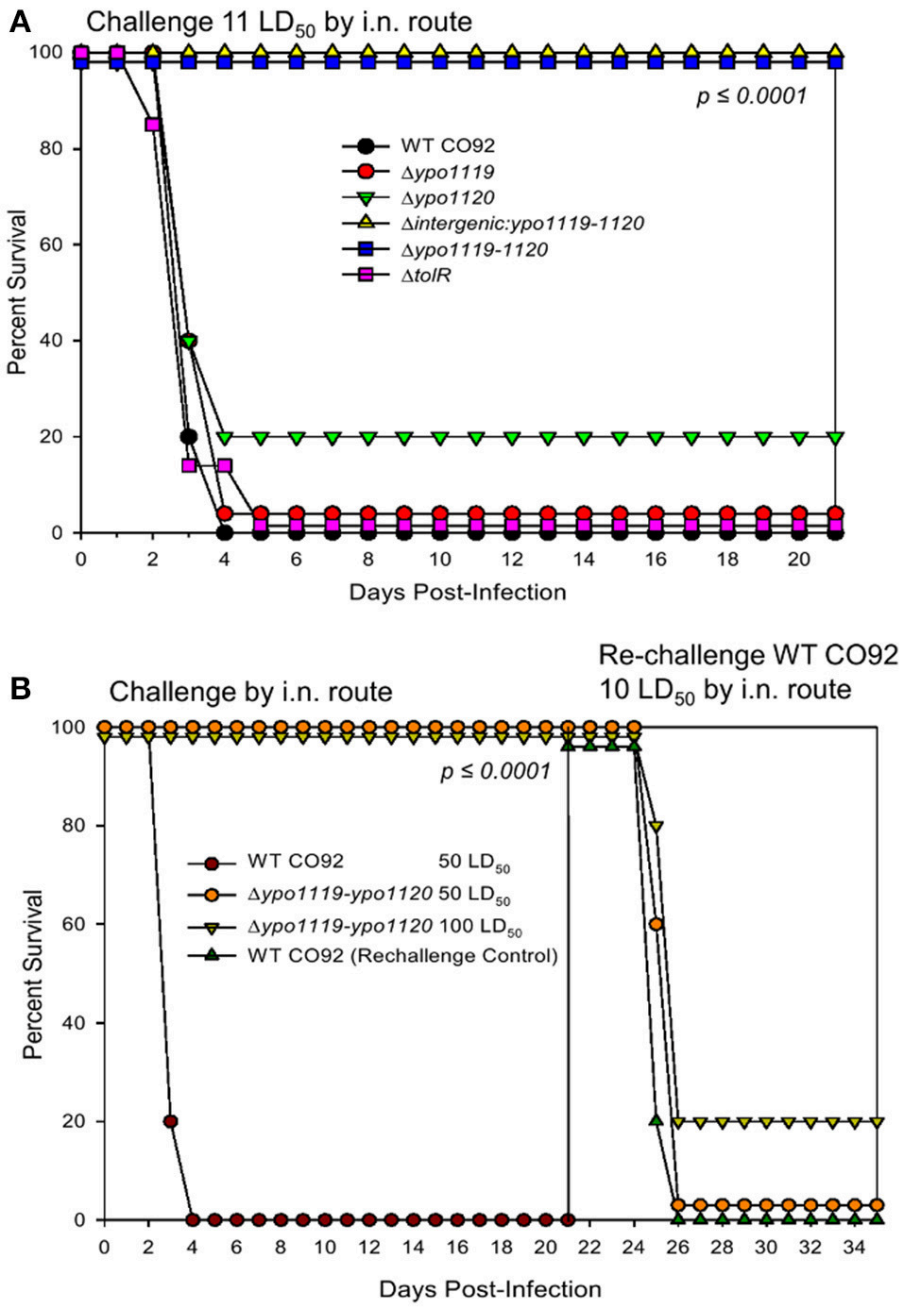
Contribution of Tol operon-associated genes in the virulence of *Y. pestis* CO92 in a mouse model of pneumonic plague. **(A)** Female Swiss-Webster mice were challenged by the i.n. route with 11 LD_50_ of WT *Y. pestis* CO92 or the indicated deletion mutant strains (*n* = 10). Animals were observed for morbidity/mortality for 21 days p.i. **(B)** Mice were challenged by the i.n. route with the WT CO92 or the Δ*ypo1119-1120* deletion mutant (*n* = 10) at the indicated LD_50_ doses and then observed for morbidity/mortality over a period of 21 days. Mice that survived the initial infection with the mutant strain (*n* = 10) and naive control animals (*n* = 5) were challenged with 10 LD_50_ of the WT CO92 strain and monitored for 14 days p.i. Survival data were analyzed for significance by Kaplan–Meier survival estimates. Two independent experiments were performed and the data from the second set constituting a group size of 10 animals/group were presented.

**Figure 3:**
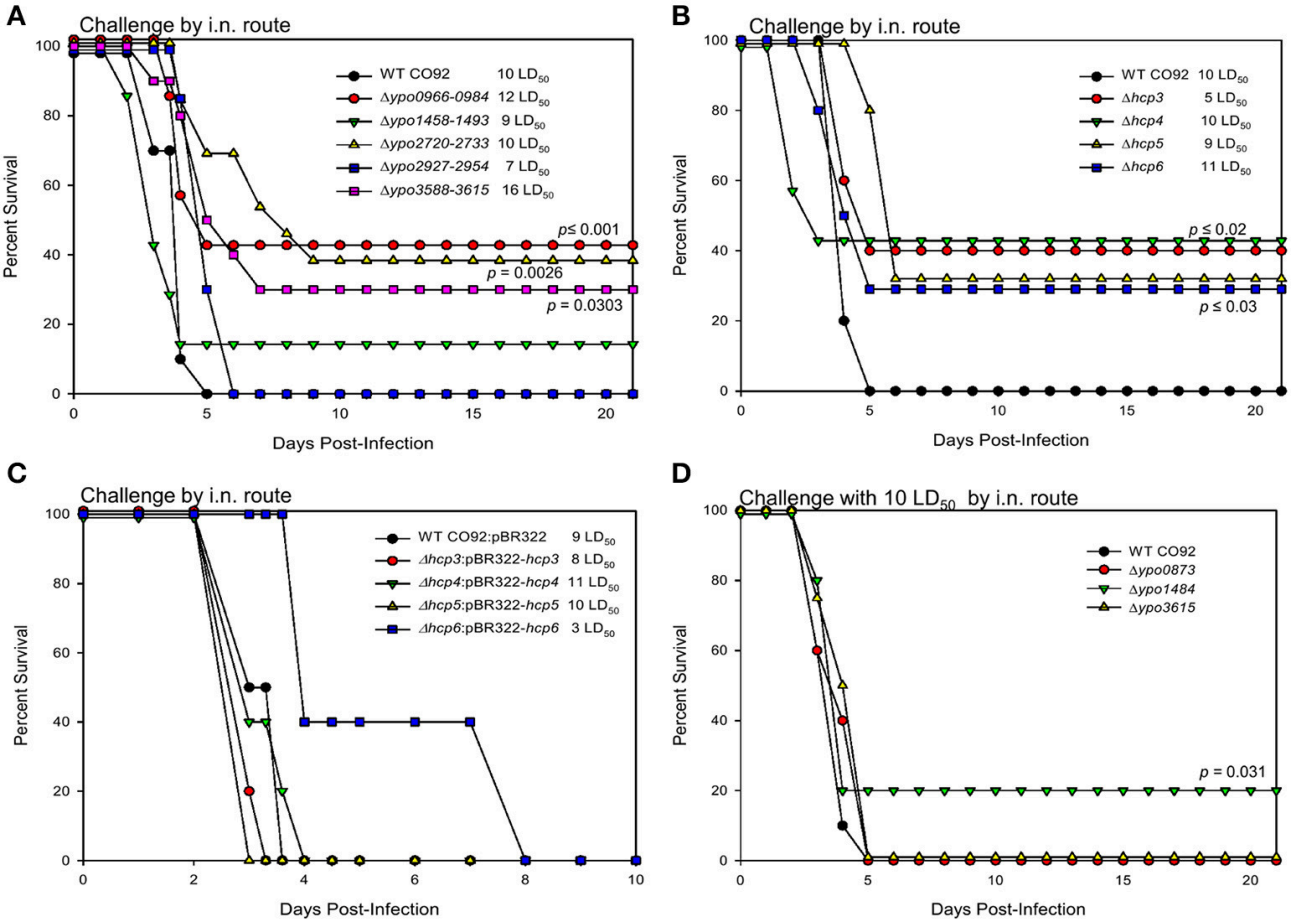
Virulence of T6SS-associated genes in a pneumonic plague mouse model. Female Swiss-Webster mice were infected with WT CO92 or 1 of the 5 T6SS cluster deletion mutant strains: Δ*ypo0966-0984*, Δ*ypo1458-1484*, Δ*ypo2720-2733*, Δ*ypo2927-2954*, Δ*ypo3588-3615* (*n* = 7–12) **(A)**, or 1 of the 4 Hcp-encoding gene deletion mutant strains: Δ*hcp3*, Δ*hcp4*, Δ*hcp5*, Δ*hcp6* (*n* = 7–12) **(B)**, or 1 of the 4 Hcp-encoding gene complemented deletion mutants strains: Δ*hcp3*:pBR322-*hcp3*, Δ*hcp4*:pBR322-*hcp4*, Δ*hcp5*:pBR322-*hcp5*, Δ*hcp6*:pBR322-*hcp6* (*n* = 5) **(C)**, or 1 of the PAAR motif-containing protein-encoding gene deletion mutants: Δ*ypo0873*, Δ*ypo1484*, Δ*ypo3615* (*n* = 10) **(D)** with the indicated LD_50_ doses by the i.n. route. Animals were observed for morbidity/mortality for 10-21 days. The data were analyzed for significance by using Kaplan–Meier survival estimates. The *P*-values were determined based on comparison of animal survival for each mutant to the survival of WT CO92-infected control animals. Our target dose of the challenge was 10 LD_50_; however, back titration of the inocula showed some variations. For the complemented *hcp6* strain, the target LD_50_ dose was 5 **(C)**. Two independent experiments were performed and the data from the second set constituting a group size of 7–12 animals/group were presented. For complemented strains and WT CO92, 5 animals/group were used as they were expected to cause lethal infection in mice.

**Figure 4:**
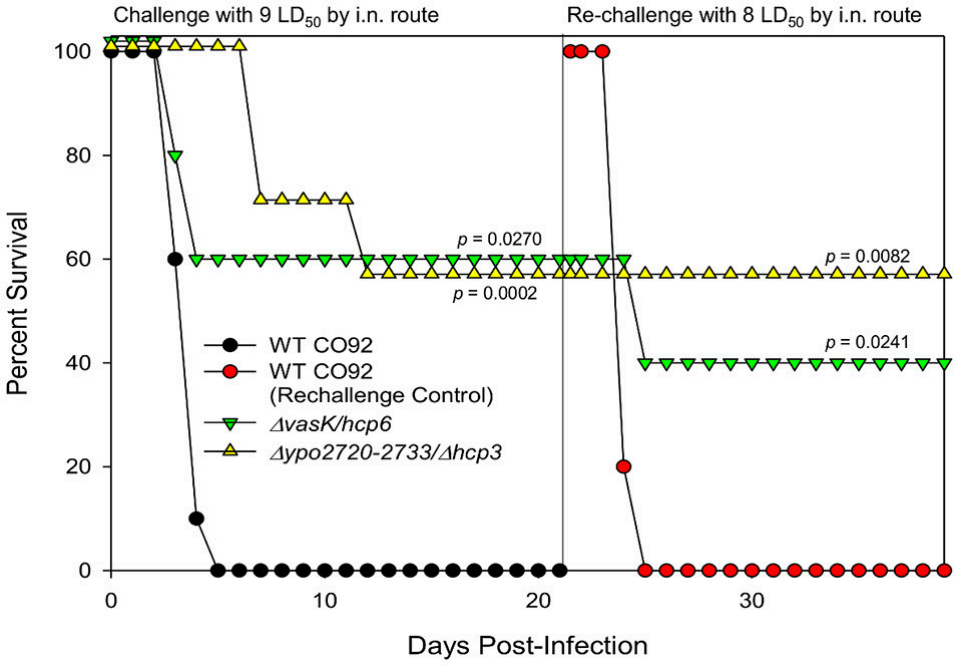
Vaccine potential for selected *Y. pestis* CO92 combinational mutants. Female Swiss-Webster mice were challenged by the i.n. route with the WT CO92, Δ*vasK*Δ*hcp6*, or Δ*2720-2733/*Δ*hcp3* (*n* = 10) at 9 LD_50_ and observed for mortality over a period of 21 days. Mice that survived the initial infection with the mutant strains and naive control animals (*n* = 5) were challenged with 8 LD_50_ of the WT CO92 strain. Survival data were analyzed for significance by Kaplan–Meier survival estimates. The *P*-values are for comparison of the results for the indicated strains to the corresponding result for WT CO92 (challenge experiment) or naive control challenged with WT CO92 (re-challenge experiment). Two independent experiments were performed and the data from the second set constituting a group size of 10 animals/group were presented.

All animal studies with *Y. pestis* were performed in an animal biosafety level 3 (ABSL-3) facility under an approved Institutional Animal Care and Use Committee (IACUC) protocol (UTMB). Six- to eight-week-old female Swiss Webster mice (17–20 g), purchased from Taconic Laboratories (Germantown, NY), were anesthetized by isoflurane inhalation and subsequently challenged either subcutaneously (s.c.), to mimic bubonic plague, or intranasally (i.n.), to mimic pneumonic plague infection, with the indicated (as shown in Figures 1–**4**) strains and LD_50_ doses (1 LD_50_ = 50 CFU by the s.c. route; 1 LD_50_ = 500 CFU by the i.n. route; Van Lier et al., 2014). Animals (*n* = 5–12) were assessed for clinical symptoms, morbidity and/or mortality for the duration of each experiment; up to 25 days post-infection (p.i.). Two independent animal experiments were performed, and all the mutants were first tested in a group of 5 animals to gauge attenuation level followed by the second experiment with 7–12 animals/group.

For re-challenge experiments, after 21 days p.i., which has been reported to be sufficient for the generation of protective antibodies against *Y. pestis* (Van Lier et al., 2014; Tiner et al., 2015a,b), the surviving mice originally infected with selected mutant strains and a group of naïve control mice (*n* = 5) were infected with WT CO92 by the i.n. route at a dose of 8–10 LD_50_ (Van Lier et al., 2014). Mice were assessed for morbidity and mortality, as well as clinical symptoms, for the duration of each experiment.

### Complementation of attenuated *hcp* homolog deletion mutant strains

Using primers 31, 34, 37, and 40 (Table S2), the complete open reading frame (ORF) of the gene of interest, along with 200 bp of the upstream DNA sequence corresponding to the promoter region of that gene, was PCR amplified with genomic DNA of WT CO92 as the template. Then, the amplified DNA constructs were cloned into plasmid pBR322 in place of the tetracycline resistance (Tc^r^)-conferring gene (Table S1; Galindo et al., 2010). The recombinant pBR322 vector was electroporated in corresponding *Y. pestis* CO92 mutants to restore the functionality of the deleted genes.

### Generation of recombinant Hcp6 protein

The *hcp6* gene (*ypo3708*, residing relatively close to T6SS Cluster G) of *Y. pestis* CO92, (Table 1) with the highest homology to *hcp* of *A. dhakensis* (82%), was cloned in the pET-30a vector to produce recombinant protein from *E. coli* as previously described (Suarez et al., 2010). The rHcp6 containing a 6 × His tag was purified by nickel affinity chromatography, dialyzed against phosphate-buffered saline (PBS), and then passed through a polymyxin B column (Bio-Rad, Hercules, CA) to remove any residual lipopolysaccharide (LPS) (Suarez et al., 2010). The pass-through fraction was filtered by using a 0.2 μm filter, and the protein concentration quantified by using the Bradford assay (Bio-Rad). The purity of rHcp6 was verified by Coomassie Blue and SYPRO-Ruby staining of the gels.

### Western blot analysis for detecting the T6SS effector hcp6 and evaluating functionality of the T3SS from *Y. pestis* CO92 mutant strains

Overnight cultures of CO92 strains, grown in HIB at 28°C, were diluted 1:20 in 5 mL HIB, supplemented with 5 mM EGTA to trigger the low-calcium response, and incubated at 28°C for 2 h before being shifted to 37°C for an additional 3 h of growth (to activate the T3SS). Bacterial cells and supernatants were separated by centrifugation. Cell pellets were dissolved in SDS-PAGE buffer and analyzed by immunoblotting using antibodies to Yop (*Yersinia* outer membrane protein) E. For supernatants, 1 mL aliquots were precipitated with 20% trichloroacetic acid (TCA, v/v) on ice for 2 h before being dissolved in SDS-PAGE buffer.

The rHcp6 of *Y. pestis* CO92 (as purified above) was used to raise polyclonal antibodies in mice for immunoblot analysis. Both cell pellets and supernatants from WT CO92 and its various mutant strains were then analyzed by immunoblotting using antibodies to Hcp6. The anti-DnaK monoclonal antibody (Enzo Life Sciences, Albany, NY) was employed for analysis of cell pellets as a loading control.

### Assessing growth kinetics, cytotoxicity on host cells, phagocytosis, and intracellular survival of *Y. pestis* CO92 strains in RAW 264.7 murine macrophages

For growth kinetics, overnight cultures of *Y. pestis* CO92 strains, grown in HIB at 28°C, were normalized to the same absorbance by measuring the optical density at 600 nm (OD_600_). Subcultures were then inoculated into 20 mL of HIB contained in 125 mL polycarbonate Erlenmeyer flasks with HEPA-filtered tops. The cultures were incubated at 28 or 37°C with agitation, and samples for absorbance measurements were taken at the indicated time points.

Viability of murine RAW 264.7 macrophages following infection was used to assess cytotoxicity of *Y. pestis* strains. Briefly, RAW 264.7 cells were seeded in 96-well microtiter plates at a concentration of 2 × 10^4^ cells/well to form confluent monolayers in a volume of 100 μL per well. *Y. pestis* strains were grown in HIB overnight as previously described. Plates were then infected with WT CO92 or the various mutant strains at a multiplicity of infection (MOI) of 100, centrifuged, and incubated at 37°C/5% CO_2_ for 60 min. Infected macrophages were then washed with PBS, treated with gentamicin (50 μg/ml) for 60 min to kill extracellular bacteria, washed again with PBS, and maintained in DMEM with a 10 μg/ml concentration of gentamicin for 12 h at 37°C/5% CO_2_ (Andersson et al., 2016). Reduction of MTT [3-(4, 5-dimethylthiazolyl-2)-2, 5-diphenyltetrazolium bromide] was used as an index of cell viability following the protocol outlined by ATCC. Briefly, MTT reagent was added to the microtiter plate wells (10 μL/well), and cells were incubated at 37°C/5% CO_2_ for an additional 2 h. Then, 100 μL of the detergent reagent was added to the wells and incubated in the dark at ambient temperature for 2 h. Absorbance values were measured at 570 nm in an ELx800 absorbance reader (Biotek, Winooski, VT).

Phagocytosis and intracellular survival of *Y. pestis* strains were determined as previously described (Sha et al., 2008; Tiner et al., 2015a). In brief, *Y. pestis* strains were grown in HIB overnight to saturation at 28°C. RAW 264.7 macrophages were seeded in 24-well plates at a concentration of 5 × 10^4^ cells/well for confluency. Macrophages were then infected with WT CO92 or the various mutant strains at a MOI of 10 in DMEM, centrifuged, and incubated at 37°C/5% CO_2_ for 60 min. Infected macrophages were then washed with PBS, treated with gentamicin (50 μg/ml) for 60 min, washed again with PBS, and maintained in DMEM with gentamicin at a concentration of 10 μg/mL until lysed for bacterial enumeration. For phagocytosis, macrophages were lysed immediately following gentamicin treatment, designated 0 h p.i., while macrophages were lysed 4 h p.i. (4 h post gentamicin treatment) for intracellular survival. The surviving bacteria inside the macrophages were assessed by serial dilution and plating on SBA plates (Sha et al., 2008; Tiner et al., 2015a). The MOIs chosen for cytotoxicity vs. phagocytosis/intracellular survival of bacteria in macrophages were empirically determined to prevent false positive results.

### Statistical analyses

Kaplan–Meier survival estimates were used for statistical analysis of animal studies. Two independent experiments were performed and data from the second experiment with animal group sizes of 7–12 (based on power analysis) were used for statistical analysis. For *in vitro* studies, two independent experiments were performed in duplicate, with the exception of cytotoxicity studies which were performed in quadruplicate. Whenever appropriate, one-way analysis of variance with Tukey's test *post-hoc* or the student's *t*-test was employed for data analysis. *P*-values of ≤ 0.05 were considered significant for all statistical tests used.

## Results

### In-frame deletion mutants *ypo0815, ypo2884*, and *cyoABCDE* of *Y. pestis* CO92 exhibited varying levels of attenuation in mice, which was further augmented when these genes were deleted from the Δ*lpp* background strain of CO92

We previously reported, through our STM screen, that transposon mutants with inserts in the genes *ypo0815, ypo2884*, and *ypo3164*, were highly attenuated in either a mouse model of bubonic plague or in a pneumonic model (Ponnusamy et al., 2015). While *ypo0815* encodes GspE of the T2SS, the *ypo2884*-encoded protein has homology to the βγ crystallin superfamily, and *ypo3164* codes for the cytochrome *o* ubiquinol oxidase subunit II, a component of the cytochrome o oxidase *(ypo3164-3168; cyoABCDE)* operon. Since transposon mutagenesis can lead to polar effects, we prepared in-frame deletion mutants of the *ypo0815* and *ypo2884* genes individually and the entire *cyoABCDE* operon to verify their role(s) in *Y. pestis* virulence.

As shown in Figure 1A, while all control WT CO92-challenged mice (at 18 LD_50_) succumbed to infection in a bubonic plague model by day 9, the above-mentioned mutants had varying levels of attenuation (20–60% survivability) at the same WT CO92 equivalent LD_50_s. However, in a pneumonic plague model, only the Δ*cyoABCDE* mutant exhibited any attenuation (30% survival) at 11 LD_50_ equivalent of WT CO92, while the other two mutants behaved like that of WT CO92 strain (Figure 1B). As we have previously shown that deletion of the *lpp* gene attenuated WT CO92 strain in mouse models of pneumonic and bubonic plague (up to 3 LD_50_; Sha et al., 2008, 2013a), we generated Δ*lpp*Δ*ypo0815*, Δ*lpp*Δ*ypo2884*, and Δ*lpp*Δ*cyoABCDE* double deletion mutants to determine whether additive or synergistic attenuation could be achieved in a pneumonic mouse model. For all three of the double deletion mutants, infected mice had statistically significant and synergistic survivability rates (50–90%) in comparison to WT CO92 control and all corresponding single gene deletion mutant-infected mice with 11 LD_50_ equivalent of WT CO92 challenge dose (Figure 1B). Animals that survived initial infection with these double deletion mutants (Δ*lpp*Δ*ypo0815*, Δ*lpp*Δ*ypo2884*, and Δ*lpp*Δ*cyoABCDE*) were then re-challenged 21 days p.i. with WT CO92 (10 LD_50_) by the intranasal (i.n.) route to evaluate the protective potential of these mutant strains. While 20–55% survival rates were observed, only animals originally infected with the Δ*lpp*Δ*cyoABCDE* mutant showed statistically significant protection from subsequent challenge with WT CO92, with all naïve control animals succumbing to infection by day 5 p.i. (Figure 1C).

### In-frame deletion of genes from the *ypo1119-1120* locus and their effects on *Y. pestis* CO92 virulence

As we previously reported from our STM screen, a mutant with transposon insertion in the intergenic region (131 bp) between *ypo1119-1120* genes exhibited significant attenuation in both bubonic and pneumonic models of plague (Ponnusamy et al., 2015). Based on analysis of WT CO92 genome, the *ypo1119-1120* genes are adjacent to the Tol-Pal system. It is also noted that the last nucleotide of *ypo1120* overlaps with the first nucleotide of *tolQ* (Figure S1A). Therefore, to further characterize the genes involved in the observed attenuation, we generated in-frame deletion mutants of Δ*ypo1119*, Δ*ypo1120*, Δ*tolQ* (*ypo1121*), Δ*tolR* (*ypo1122*), and Δ*intergenic:ypo1119-ypo1120*, as well as the *ypo1119-1120* entire locus deletion mutant, Δ*ypo1119-1120*. During *in vitro* assays, it was observed that growth of Δ*tolQ* mutant was generally hindered, to a point of statistical significance during the later stages, at 28°C, compared to WT CO92 (Figure S1B). At 37°C, the growth of Δ*tolQ* mutant was completely inhibited (Figure S1C), and therefore, was not evaluated in an *in vivo* model of infection. It was also noted that growth of Δ*intergenic:ypo1119-ypo1120* and Δ*ypo1119-1120* mutants was reduced in comparison to WT CO92 during late growth stages at 37°C (Figure S1C).

The above mutants were then further evaluated in a mouse model of pneumonic plague. As observed in Figure 2A, the Δ*intergenic:ypo1119-ypo1120* and Δ*ypo1119-1120* deletion mutants were 100% attenuated at a dose equivalent to 11 LD_50_ of WT CO92. However, at the same dose range, the Δ*ypo1119* single mutant infected mice exhibited only 20% survivability and the Δ*ypo1120* and Δ*tolR* single mutants exhibited a phenotype similar to that of WT CO92, with all animals succumbing to infection by day 4. The Δ*ypo1119-1120* mutant strain was then further evaluated for attenuation and vaccine potential. Animals were challenged with doses equivalent to 50 and 100 LD_50_ of WT CO92 by the i.n. route. As shown in Figure 2B, both doses of the mutant strain resulted in 100% survivability of the animals. The surviving animals, 21 days p.i., were infected with WT CO92 by the i.n. route with a 10 LD_50_ dose. Following re-challenge, all animals originally infected with 50 LD_50_ of Δ*ypo1119-1120* mutant succumbed to infection, while 80% of them originally challenged with 100 LD_50_ of Δ*ypo1119-1120* succumbed to infection (Figure 2B). Thus, this mutant did not generate protective immunity in animals.

### Deletion of T6SS clusters and effectors resulted in varying levels of attenuation in a pneumonic plague mouse model

Using STM approach, we have previously reported the identification of three T6SS genes with virulence potential in *Y. pestis* CO92 (Ponnusamy et al., 2015). Indeed, the contribution of the T6SS to *Y. pestis* virulence was confirmed through generation of Δ*vasK* and Δ*lpp*Δ*vasK* deletion mutants, which resulted in significant attenuation of the bacterium in murine models of infection (Ponnusamy et al., 2015). Analysis of the CO92 genome revealed six T6SS clusters (Boyer et al., 2009; Robinson et al., 2009), six Hcp protein-encoding gene homologs, and five PAAR motif-containing protein-encoding genes (Table 1). Hcp is a well-established structural component as well as an effector and marker of functional T6SS, while PAAR motif-containing proteins have been identified to form the spike complex along with valine glycine rich G family proteins (VgrG) to penetrate target host membranes to translocate/secrete effectors (Filloux, 2013). One T6SS cluster (Cluster A, *ypo0498-0518*) was previously shown by our laboratory to have no effect on *Y. pestis* virulence *in vivo* (Robinson et al., 2009), while the other five were previously uncharacterized. In this study, we have shown generation of these five T6SS cluster (B, C, E–G) deletion mutants (Table 1) resulted in varying levels of attenuation when evaluated in a mouse model of pneumonic plague (Figure 3A). Deletion mutants for Cluster C (*ypo1458-1484*) and Cluster F (*ypo2927-2954*) exhibited limited, 14%, or no attenuation, respectively, while deletion mutants for Cluster B (*ypo0966-0984*), partial Cluster E (*ypo2720-2733*), and Cluster G (*ypo3588-3615*), exhibited significant levels of attenuation, 30–42%, in comparison to WT CO92 (Figure 3A).

For the 6 *hcp* homologs, two (*ypo0973* and *ypo1470*; designated as *hcp1* and *hcp2*) were contained within the T6SS clusters B and C, respectively, while the other four were located outside of the identified T6SS clusters (Table 1). Consequently, only the four Hcp-encoding genes outside of these T6SS clusters had deletion mutants generated, as any effect resulting from *hcp1* or *hcp2* deletion would be observed through its corresponding whole T6SS cluster (B or C) deletion. All Hcp-encoding gene deletion mutants were observed to have statistically significant levels of attenuation, between 30 and 42%, compared to WT CO92 (Figure 3B). To further confirm that attenuation was a direct result of deletion of the *hcp* homolog genes, mice were challenged with the complemented strains, Δ*hcp3*:pBR322-*hcp3*, Δ*hcp4*:pBR322-*hcp4*, Δ*hcp5*:pBR322-*hcp5*, and Δ*hcp6*:pBR322-*hcp6*. Animals challenged with these strains all succumbed to infection in a pneumonic model. Although, a somewhat delayed mean time to death was observed in some of the complemented strains, specifically the Δ*hcp6*:pBR322-*hcp6* mutant (even at a much lower challenge dose of 3 LD_50_), this delay did not reach statistical significant as compared to WT CO92. Thus, complementation of the *hcp* genes resulted in restoration of the WT phenotype (Figure 3C).

Of the identified PAAR motif-containing protein-encoding genes, three of the five had deletion mutants generated: Δ*ypo0873*, Δ*ypo1484*, and Δ*ypo3615*. Of these mutants, only Δ*ypo1484* exhibited a limited level of attenuation, 20% survivability, in comparison to WT CO92 (Table 1; Figure 3D). This attenuation was similar to the level of attenuation, 14%, reported in the T6SS Cluster C (Δ*ypo1458-1484*) deletion mutant, which contains the *ypo1484* gene (Table 1; Figure 3A).

### Generation of combinatorial T6SS deletion mutants further augmented attenuation in a murine model of pneumonic plague and provided protection against re-challenge with WT CO92

As single T6SS cluster and single *hcp* homolog deletion mutants exhibited 14–42% attenuation *in vivo* (Figures 3A,B), we next evaluated whether this attenuation could be augmented through the generation of combinatorial deletion mutants. As we have previously reported Δ*vasK* to be attenuated and *hcp6* has the highest homology (82%) to Hcp-encoding genes in other bacterial species, such as *A. dhakensis* and *V. cholerae* (Pukatzki et al., 2006; Suarez et al., 2010; Ponnusamy et al., 2015), we generated a Δ*vasK*Δ*hcp6* double deletion mutant. Additionally, we generated a Δ*ypo2720-2733*Δ*hcp3* (Cluster E and *hcp* homolog 3) double deletion mutant. When evaluated for attenuation in a mouse model of pneumonic plague at 9 LD_50_ dose equivalent of WT CO92, both double deletion mutants exhibited significantly high levels of attenuation, 60%, in comparison to WT CO92 (Figure 4). This attenuation was also observed to be additive for both double deletion mutants as their single gene deletion counterparts exhibited 20–40% survival (Figures 3A,B; Ponnusamy et al., 2015). It was also observed that in animals that did succumb to infection when challenged with the Δ*ypo2720-2733*Δ*hcp3* mutant, there was an increased time to death (day 7–12 vs. day 3–5).

As one of our long-term goals is to develop live-attenuated vaccine candidates, animals that survived initial infection with Δ*vasK*Δ*hcp6* and Δ*ypo2720-2733*Δ*hcp3* mutants were re-challenged with WT CO92 by the i.n. route to mimic pneumonic infection. As shown in Figure 4, 100% of the animals initially infected with Δ*ypo2720-2733*Δ*hcp3* and re-challenged with WT CO92 at 8 LD_50_ survived and were observed to be protected over a tested period of 18 days. For the Δ*vasK*Δ*hcp6* mutant, one animal did succumb to infection; however, still exhibited significant, 40%, survivability in comparison to control WT CO92 challenged mice, which all succumbed to infection by day 4 (Figure 4). Therefore, these mutants can serve as background strains for the deletion of additional genes to further attenuate the bacterium while maintaining immunogenicity.

### Characterization of growth kinetics and expression of T3SS and T6SS effectors from the attenuated T6SS mutant strains *in Vitro*

Strains significantly attenuated in *in vivo* studies were further evaluated *in vitro* to begin to elucidate potential mechanisms of attenuation. We first evaluated growth kinetics of the attenuated mutant strains, Δ*ypo0966-0984*, Δ*ypo2720-2733*, Δ*ypo3588-3615*, Δ*hcp3*, Δ*hcp4*, Δ*hcp5*, Δ*hcp6*, Δ*vasK*, Δ*vasK*Δ*hcp6*, and Δ*ypo2720-2733*Δ*hcp3, in vitro* at 28 and 37°C, to mimic both flea and eukaryotic host temperatures. At both 28 or 37°C, none of the mutants were observed to have any growth defects in comparison to WT CO92 (Figures S2, S3).

**Figure 5:**
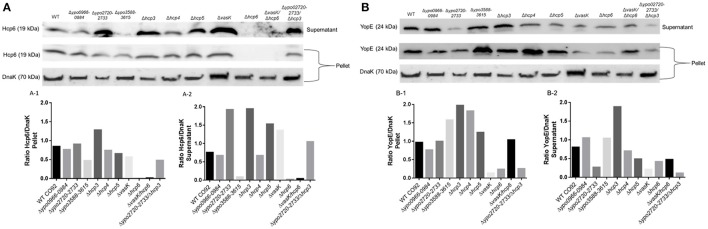
Production of the T6SS effector Hcp6 and the T3SS effector YopE. WT *Y. pestis* CO92 and the indicated T6SS mutant strains were grown in HIB overnight and subsequently diluted 1:20 in fresh HIB supplemented with 5 mM EGTA. Growth was continued at 28°C for 2 h, followed by a temperature shift to 37°C for an additional 3 h of incubation. Bacterial pellets and supernatants were dissolved in SDS-PAGE buffer. Western blot analysis was then performed to detect Hcp6 **(A)** and YopE **(B)** in bacterial pellet and supernatant samples. Levels of DnaK in bacterial pellets were utilized as a loading control to ensure similar levels of bacteria were used across all samples examined. Sizes of the proteins are indicated in parentheses. Densitometry scanning was performed by using Image Studio^TM^ Lite Software (Li-Cor Inc., Lincoln, NE) to quantitate expression of Hcp6 and YopE in ratio to DnaK in the pellet **(A-1, B-1)** and supernatant **(A-2, B-2)** samples. The same blot was used for analyzing both Hcp6 and YopE as well as DnaK. The experiment was performed twice and typical data from one of the experiments is presented.

Hcp secretion from bacteria is a well-established marker of a functional T6SS (Pukatzki et al., 2006). In order to confirm that deletion of *hcp6* alone or in combination with *vasK*, as well as if any other deletion attenuated mutants had an effect on this effector, Hcp6 production and secretion was evaluated by Western blot analysis (Figure 5A). Except for mutants Δ*hcp6* and Δ*vasK/hcp6* which served as negative controls, Hcp6 was detected in the pellet fractions of WT CO92 and all other mutant strains, with slightly increased level in the Δ*hcp3* mutant (Figure 5A-1). However, Hcp6 secretion differed in several mutant strains (Figure 5A-2). In comparison to WT CO92, mutants Δ*ypo2720-2733*, Δ*hcp3*, Δ*hcp5*, Δ*vasK*, and Δ*ypo2720-273/*Δ*hcp3* exhibited increased secretion of Hcp6 (Figure 5A-2). The above data indicated that several of these genes did effect Hcp6 secretion in *Y. pestis*, and deletion of the *hcp3* gene affected both expression and secretion levels of Hcp6. Interestingly, for the Δ*ypo3588-3615* mutant, production of Hcp6 was noted in the pellet fraction, but exhibited no secretion of Hcp6 in the supernatant fraction (Figures 5A-1,A-2). These data indicated that Hcp6 is secreted through T6SS Cluster G. Anti-Hcp6 antibodies did not cross-react with other Hcp homologs because of low homologies (31–34%; Table 1).

As the T3SS is an essential and well-studied virulence mechanism of *Y. pestis*, we wanted to determine if deletion of any T6SS components affected T3SS functionality. Production of YopE, a well characterized component of the T3SS which destroys actin monofilaments, was evaluated by Western blot analysis in attenuated mutant strains and WT CO92. YopE production differed in several mutants in both bacterial supernatant and pellet fractions (Figures 5B-1,B-2). While all mutants did produce YopE to some extent in pellet fractions, mutants Δ*ypo3588-3615*, Δ*hcp3*, and Δ*hcp4* exhibited increased production while mutants Δ*vasK*, Δ*hcp6*, and Δ*ypo2720-2733/*Δ*hcp3* exhibited decreased production (Figure 5B-1). In terms of YopE secretion, several mutants exhibited decreased production in supernatant fractions in comparison to WT CO92, apart from mutants Δ*ypo0966-0984* and Δ*ypo3588-3615*, with expression levels comparable to WT CO92, and Δ*hcp3*, with expression of YopE higher in comparison to WT CO92 (Figure 5B-2). These data indicated a potential interplay between these T6SS-associated genes and the T3SS.

### Role of T6SS in host cell cytotoxicity following *Y. pestis* infection

**Figure 6:**
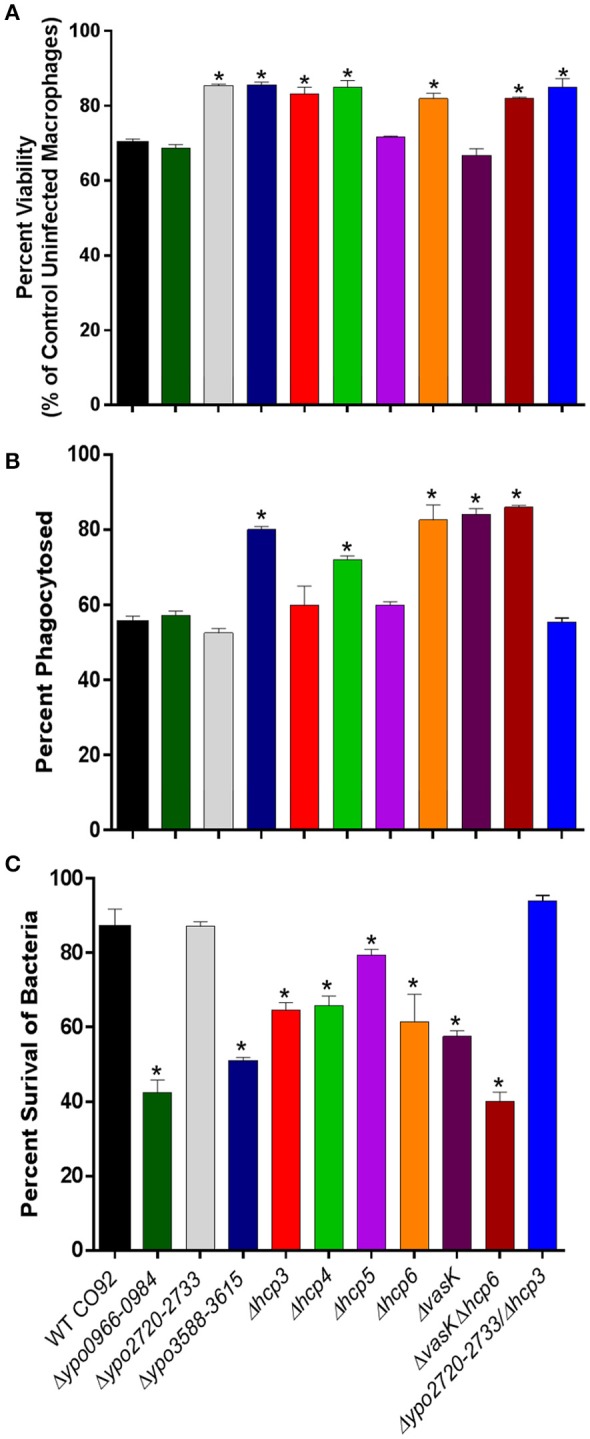
Possible mechanisms of attenuation of *Y. pestis* CO92 mutants**. (A)** Determination of bacterial cytotoxicity to murine macrophages. RAW264.7 murine macrophages were infected with the indicated *Y. pestis* strains at an MOI of 100 for 1 h, washed with PBS, treated with gentamicin (50 μg/ml) for 1 h, then washed with PBS and incubated with a maintenance dose of gentamicin (10 μg/ml) in DMEM for 12 h. Viability following infection was then evaluated by the MTT assay. The data were plotted as mean percentages of uninfected macrophages, pooled from two independent experiments, with four replicates per experiment, and were analyzed by one-way ANOVA with Tukey's *post-hoc* test. ^*^P < 0.05. **(B)** Percent phagocytosis of *Y. pestis* strains in RAW 264.7 murine macrophages. Macrophages were infected with the indicated *Y. pestis* strains at an MOI of 10 for 1 h, and after gentamicin treatment and wash, were lysed for bacterial enumeration (0-h sample for phagocytosis). CFUs were determined by serial dilution and plating on SBA plates. Percent of phagocytosed bacteria was calculated based on the number of bacteria used to infect the macrophages. Two independent experiments in duplicate (for a total *n* = 4) were performed. The data were analyzed by comparison of phagocytosed WT CO92 to mutant strains using one-way ANOVA with Tukey's *post-hoc* test. ^*^P ≤ 0.001. **(C)** Quantification of intracellular survival of *Y. pestis* CO92 mutant strains in RAW 264.7 murine macrophages. RAW 264.7 macrophages were infected with *Y. pestis* CO92 strains at an MOI of 10. At 4 h p.i. (after gentamicin treatment), macrophages were lysed and CFUs determined by serial dilution and plating on SBA plates. Two independent experiments in duplicate (for a total *n* = 4) were performed. The data were analyzed by comparison of mutant strains to WT CO92 using one-way ANOVA with Tukey's *post-hoc* test. ^*^P < 0.01.

The T6SS delivers effector molecules into target cells using a needle like apparatus with high homology to the assembly, structure, and function of bacteriophage tails (Records, 2011). It has been reported that T6SS can target both bacterial and eukaryotic cells and cause cytotoxicity (Russell et al., 2014). To evaluate any changes in cytotoxic effects of the attenuated T6SS deletion mutant strains on eukaryotic cells, we used the MTT assay to measure cell viability following infection. RAW 264.7 murine macrophages were infected with the various *Y. pestis* strains (WT CO92 or the Δ*ypo0966-0984*, Δ*ypo2720-2733*, or Δ*ypo3588-3615* clusters, Δ*hcp3*, Δ*hcp4*, Δ*hcp5*, Δ*hcp6*, Δ*vasK* single, or Δ*vasK*Δ*hcp6* and Δ*ypo2720-2733*Δ*hcp3* double deletion mutants) at a MOI of 100, and incubated for 12 h. Macrophages infected with two cluster deletion mutants (Δ*ypo2720-2733* and Δ*ypo3588-3615*), three *hcp* homolog deletion mutants (Δ*hcp3*, Δ*hcp4*, and Δ*hcp6*), and both combinatorial deletion mutants (Δ*vasK*Δ*hcp6* and Δ*ypo2720-2733*Δ*hcp3*) exhibited significantly higher cell viabilities in comparison to WT CO92 (Figure 6A). These results indicated that the above-mentioned mutant strains were significantly less cytotoxic to host cells during infection in comparison to WT CO92.

### Quantification of phagocytosis and intracellular survival of T6SS deletion mutant strains

The attenuated T6SS deletion mutants identified *in vivo* (Δ*ypo0966-0984*, Δ*ypo2720-2733*, Δ*ypo3588-3615*, Δ*hcp3*, Δ*hcp4*, Δ*hcp5*, Δ*hcp6*, Δ*vasK*, Δ*vasK*Δ*hcp6*, and Δ*ypo2720-2733*Δ*hcp3*), were evaluated for any significant changes in phagocytosis in comparison to WT CO92 in RAW 264.7 murine macrophages. For the cluster deletion mutants, including the double deletion mutant Δ*ypo2720-2733*Δ*hcp3*, only Δ*ypo3588-3615*, which includes *vasK* (*ypo3603*), exhibited any significant increase in phagocytosis (Figure 6B). For the *hcp* homolog deletion mutants, Δ*hcp4* and Δ*hcp6* exhibited significant increases in phagocytosis in comparison to WT CO92, although it was noted that the increase observed for Δ*hcp4* was not to the extent of Δ*hcp6* (*p* = *0.022* for Δ*hcp6* vs. Δ*hcp4*). Additionally, both the Δ*vasK* and Δ*vasK*Δ*hcp6* double mutant exhibited significantly increased rates of phagocytosis.

We next evaluated the ability of the various attenuated T6SS deletion mutants to survive in RAW 264.7 murine macrophages to determine the role these genes may play in intracellular survival of *Y. pestis*. Macrophages were infected with WT CO92 or the Δ*ypo0966-0984*, Δ*ypo2720-2733*, and Δ*ypo3588-3615* clusters, Δ*hcp3*, Δ*hcp4*, Δ*hcp5*, Δ*hcp6*, Δ*vasK* single, or Δ*vasK*Δ*hcp6* and Δ*ypo2720*-*2733*Δ*hcp3* double deletion mutants at a MOI of 10. The evaluated strains all exhibited significantly lower intracellular survival at 4 h p.i. in comparison to WT CO92, with the exception of 2 strains; Δ*ypo2720-2733* and Δ*ypo2720-2733*Δ*hcp3* (Figure 6C).

**Figure 7:**
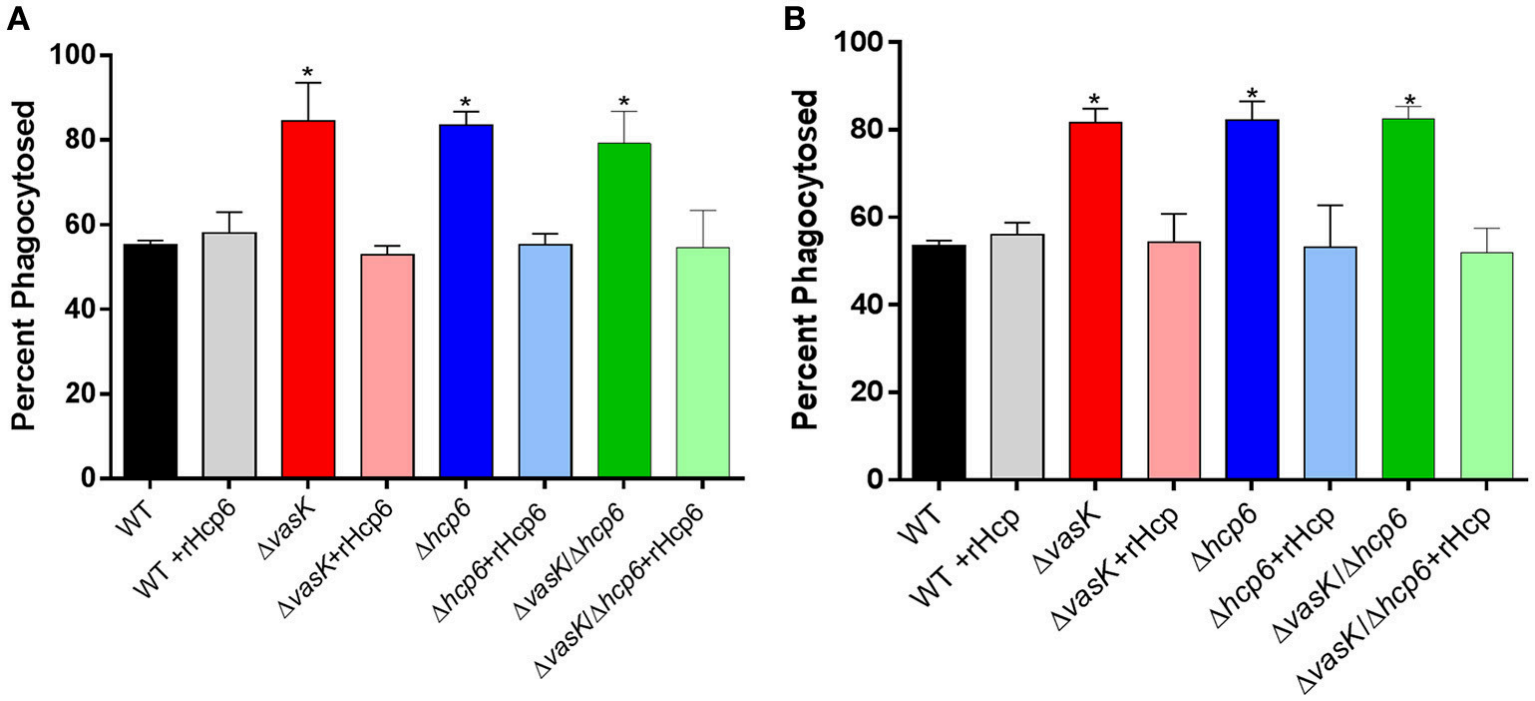
Phagocytosis of WT CO92, Δ*vasK*, Δ*hcp6*, and Δ*vasK*Δ*hcp6* mutants in RAW264.7 murine macrophages with presence of exogenous recombinant Hcps. Macrophages were infected with WT and the indicated mutant strains at MOI of 10 and incubated for 1 h with or without 10 μg/ml rHcp6 [*Y. pestis* **(A)**] or rHcp [*A. dhakensis* **(B)**]. Cells were then washed and incubated for 1 h with gentamicin to eliminate extracellular bacteria before being lysed for intracellular bacterial enumeration (see legend for Figure 6). Two independent experiments with two replicates (for a total *n* = 4) were performed. The data were analyzed by comparison of each strain with/out the addition of rHcp6/rHcp using one-way ANOVA with Tukey's *post-hoc* test. ^*^P < 0.01. The addition of rHcp6 or rHcp alone did not affect macrophage survival (data not shown).

As we have previously reported that Hcp inhibits phagocytosis of *A. dhakensis* (Suarez et al., 2010), we further evaluated the phagocytic role of *hcp6*, which has high homology (82%) to *hcp* of *A. dhakensis*, in *Y. pestis*. RAW 264.7 murine macrophages were infected with WT CO92, Δ*vasK*, Δ*hcp6*, or Δ*vasK*Δ*hcp6* mutant strains with or without the addition of recombinant(r)Hcp6 from CO92 (Figure 7A) or rHcp from *A. dhakensis* (Figure 7B), thus allowing for complementation of the mutants with exogenous Hcp6 protein. We noted that for all three mutant strains tested, the rate of phagocytosis was significantly increased in comparison to WT CO92 (Figures 7A,B). Furthermore, the ability of these mutants to be phagocytosed was significantly reduced, to levels equivalent to that of WT CO92, with the addition of rHcp6/rHcp at a physiologically relevant concentration of 10 μg/mL (Suarez et al., 2010; Figures 7A,B).

## Discussion

The need to develop targeted live-attenuated vaccines for plague is evident given its status as a Tier 1 select agent, a re-emerging human pathogen, and potential for use as a biological weapon (Inglesby et al., 2000; Ligon, 2006). Although, several virulence factors of *Y. pestis* have been identified and well characterized, they alone cannot account for the extremely virulent phenotype of the plague bacterium.

To identify other key virulence factors of *Y. pestis*, we recently performed a genome-wide functional study utilizing the STM approach. While we were able to identify known virulence factor encoding genes, e.g., plasminogen-activator (Pla) protease and F1, several genes encoded hypothetical proteins with unknown functions. One of the genes, *ypo0815*, has significant homology to genes of other *Enterobacteriaceae* family members encoding GspE, a conserved ATPase essential to the functioning of the T2SS (Camberg and Sandkvist, 2005; Korotkov et al., 2012; Lu et al., 2014). Studies in *Y. enterocolitica* have revealed two T2SS loci in the genome, namely Yst1 and Yst2, with Yst1 shown to play an important role in pathogen virulence through mutational inactivation in murine infection models (Iwobi et al., 2003; von Tils et al., 2012). However, *in silico* analysis revealed only one T2SS locus in the genome of *Y. pestis*, which shares homology to the Yst2 locus, whose role is largely unknown for any *Yersinia* species (von Tils et al., 2012). Generation of an in-frame deletion mutant of *ypo0815* revealed significant attenuation of *Y. pestis* in a murine model of bubonic plague (Figure 1A). With the combinatorial deletion mutant Δ*lpp*Δ*ypo0815*, synergistic attenuation was also observed in the pneumonic model of plague (Figure 1B), supporting *ypo0815*'s role in *Y. pestis* pathogenesis. Attenuation in the virulence of the Δ*ypo0815* mutant could be associated with failure to secrete bacterial enzymes/toxins through the T2SS, such as metallo-protease(s), RNase I, phospholipases, lyso-phospholipases, and acid phosphatase (which exist in WT CO92), to the extracellular milieu (Sandkvist, 2001; Li and Yang, 2008; Korotkov et al., 2012).

Our STM screen also identified *ypo2884*, which encodes a putative exported protein with homology to those of the βγ crystallin superfamily, and the transposon mutant was significantly attenuated in both bubonic and pneumonic models of plague (Ponnusamy et al., 2015). The βγ crystallin superfamily consists of evolutionary related proteins found in microbes to vertebrates (Suman et al., 2013). The presence of βγ domain proteins in several pathogenic bacteria (Suman et al., 2013) raises questions about their functions in virulence and disease pathogenesis, particularly where calcium is known to play an important role in the physiology and virulence of the pathogen, as some members of the βγ crystallin have been shown to bind calcium (Aravind et al., 2009; Srivastava et al., 2014). In *Y. pestis*, it has been predicted that *ypo2884* might play a role in the low calcium response (Jobby and Sharma, 2005).

In a previous study, both domains, D1 and D2, of the crystallin encoded by *ypo2884*, were observed to bind calcium. Calcium was observed to exert extrinsic stabilization on domain D1 and was required for the typical βγ crystallin fold structure, suggesting a role in calcium-regulated processes, such as stress response or physiology, in *Y. pestis* (Jobby and Sharma, 2005). Generation of an in-frame deletion mutant of *ypo2884* and a combinatorial Δ*lpp*Δ*ypo2884* mutant resulted in bacterial attenuation in bubonic and pneumonic mouse models of plague, respectively (Figures 1A,B), thus providing additional evidence of this protein playing a vital role in the pathogenesis of *Y. pestis* infection. However, the mechanism underlying this attenuation is unclear and requires further investigation.

Based on STM screen, several identified genes were involved in bacterial metabolism (Ponnusamy et al., 2015). One such gene was *ypo3164*, which encodes cytochrome *o* ubiquinol oxidase subunit II (CyoA). The cytochrome *o* oxidase complex is coded for by *ypo3164-3168 (cyoABCDE*) operon and is a key terminal oxidase in the aerobic respiratory chain of bacteria (Cotter et al., 1990). This system is directly coupled to the generation of cellular energy. During aerobic growth under mildly acidic conditions, an upregulation of components of the cytochrome *o* oxidase system has been observed, which suggests the coupling of proton efflux to metabolism via components of this system (Kanjee and Houry, 2013).

In both *Yersinia* species and *E. coli*, components of the cytochrome *o* oxidase system have been described to be transcriptionally regulated based on growth medium and/or temperature (Cotter et al., 1990; Rosso et al., 2008; Pettersen et al., 2016). While the differential expression of enzymes and components under differing conditions suggest that cytochrome oxidase synthesis is regulated, the implication for virulence is largely unknown. Studies in *Staphylococcus aureus* have observed a role for terminal oxidases in bacterial fitness as well as virulence, with a mutant deleted for a component of the *qoxABCD* operon, which is structurally related the CyoABCDE system, exhibiting decreased dissemination to the liver in a systemic infection model (Lan et al., 2010). To maximize the effect on bacterial virulence, we generated a Δ*cyoABCDE* operon deletion mutant to evaluate for attenuation in mouse models of *Y. pestis* infection. Although, attenuation with this mutant was limited (Figure 1A), in both bubonic and pneumonic murine models of plague infection, deletion of *cyoABCDE* in combination with that of *lpp* exhibited a synergistic level of attenuation in a pneumonic plague model (Figure 1B). Interestingly, the Δ*lpp*Δ*cyoABCDE* double deletion mutant was effective in eliciting a protective immune response (Figure 1C).

One transposon insertion during STM occurred within the intergenic region of *ypo1119-1120*, which resulted in a high level of attenuation in both bubonic and pneumonic models of plague (Ponnusamy et al., 2015). While deletion of the entire *ypo1119-1120* locus or the intergenic (131 bp) region alone resulted in 100% bacterial attenuation, surprisingly, neither the Δ*ypo1119* nor the Δ*ypo1120* single gene deletion mutant provided attenuation in a pneumonic murine model of plague (Figure 2A). Upon further analysis of the intergenic region, a small putative ORF (75 bp), previously unannotated, was identified. Interestingly, the transposon insertion interrupted the stop codon of the ORF (Ponnusamy et al., 2015; Figure S1A). However, complementation of the Δ*intergenic:ypo1119-ypo1120* mutant with the 75 bp-ORF did not alter the attenuated phenotype of the mutant (data not shown), indicating it was not coding for an independent virulence factor. Also, BLAST search revealed the 75 bp-ORF shares limited homology (32%) with the TetR family of transcriptional regulators from *Actinokineospora bangkokensis* and ArsR family of transcriptional regulators from *Proteobacteria*. Therefore, the 131 bp-intergenic region most likely serves as a promoter for the *ypo1120* gene. As the *ypo1120* gene is genetically linked to *tolQ* (Figure S1A), interrupting the intergenic region of *ypo1119-1120* may affect the Tol-Pal system, leading to the observed attenuation of this deletion mutant *in vivo*.

The Tol-Pal system is well conserved among Gram-negative bacteria and contains at least five interacting envelope proteins, namely TolQ, TolR, TolA, TolB, and Pal (Gerding et al., 2007).

This system is involved in several bacterial functions such as uptake of filamentous phage DNA, surface expression of LPS O-antigen, resistance to detergents, outer-membrane integrity and stability, cell division, and virulence (Click and Webster, 1998; Journet et al., 1999; Lazzaroni et al., 1999; Gerding et al., 2007). The *Tol- pal-* mutants of *Erwinia chrysanthemi*, a plant pathogen, display a reduced ability to grow in plant tissues and to exhibit increased susceptibility to antimicrobials (Dubuisson et al., 2005). Additionally, *tol* operon as well as *tol-pal* operon deletion mutants of *Salmonella* SL1344 were attenuated in mouse models of infection (Paterson et al., 2009). Dysregulation of cell membrane integrity results in a lack of bacterial fitness and virulence (Bernadac et al., 1998), and incidentally, a lack of bacterial fitness may lead to rapid clearance of the bacteria, before the generation of an effective immune response, which may be the case as we noted for the *ypo1119-1120* mutant (Figure 2B). Interestingly, in-frame deletion of the *tolR* gene did not result in CO92 attenuation (Figure S1A and Figure 2A). The *tolQ* in-frame deletion mutant was unable to grow at 37°C (Figures S1B,C), and, hence, was not tested in a mouse model of plague. To better understand an exact role of Tol-Pal system in the pathogenesis of *Y. pestis* infection, it is critical that an interplay among different components of this system as well as with the other virulence factors and regulatory circuits of the pathogen be further investigated.

Three genes of the T6SS were also identified in our STM screen, with the Δ*vasK* and Δ*lpp*Δ*vasK* deletion mutants showing attenuation in both bubonic and pneumonic models of plague providing the first evidence that T6SS is involved in *Y. pestis* virulence (Ponnusamy et al., 2015). Although the T6SS has been the target for potential vaccine development in *B. mallei* (Hatcher et al., 2016), its role in *Y. pestis* virulence remains mostly unknown. The *Y. pestis* genome contains several T6SS loci, which differ in both gene numbers and arrangements, and several T6SS effectors, which are contained both within and outside of these predicted T6SS clusters (Boyer et al., 2009; Robinson et al., 2009). For this study, we generated five uncharacterized T6SS cluster deletion mutants, four *hcp* homolog deletion mutants, and three PAAR motif repeat-containing protein-encoding gene deletion mutants (Table S1). The T6SS locus *ypo2715-2733* contained the gene *lepA* (*ypo2716*), which has been shown to be an essential bacterial translation factor (Qin et al., 2006), so the deletion mutant Δ*ypo2720-2733* was generated to elucidate the role of this T6SS cluster on *Y. pestis* virulence. Additionally, only 4 of the 6 *hcp* genes were targeted for deletion as the other two were encoded within two of the T6SS loci. For three cluster deletion mutants, Δ*ypo0966-0984*, Δ*ypo2720-2733*, and Δ*ypo3588-3615*, and all four *hcp* homolog deletion mutants generated, a significant attenuation of virulence (Figure 3), was observed in a murine pneumonic plague model in comparison to WT CO92. Through the generation of two combinatorial deletion mutants, Δ*vasK*Δ*hcp6* and Δ*ypo*2720-2733Δ*hcp3*, attenuation could be further augmented (Figure 4). Further, these two mutants generated protective immunity in mice (Figure 4).

Attenuation of mutant strains *in vivo* may be the result of several factors including defects in growth or defects in ability to evade and survive the host's immune response. For all T6SS-associated attenuated strains identified *in vivo*, none had any growth defects in comparison to WT CO92 when grown at both 28 and 37°C (Figures S2, S3). In evaluating T6SS functionality, specifically in terms of Hcp6 production and secretion, as expected, no Hcp6 production was noted in Δ*hcp6* or Δ*vasK/*Δ*hcp6* mutant strains (Figures 5A-1,A-2). Interestingly, for Δ*ypo3588-3615* mutant, Hcp6 was not secreted in the supernatant fraction, but was found in the bacterial pellet fraction. These results indicated that Hcp6 could be secreted through the *ypo3588-3615* locus.

As T3SS is a well-established virulence factor of *Y. pestis* (Cornelis, 2002), we also evaluated this system's functionality in the attenuated mutant strains (Figures 5B-1,B-2). For YopE expression, several mutants showed varying production levels in comparison to WT CO92. In *P. aeruginosa*, studies have shown a link between regulation of the T3SS and T6SS, with evidence presented that c-di-GMP levels can modulate the switching of these secretion systems (Moscoso et al., 2011). While our results may suggest a similar link in *Y. pestis*, future studies looking more into this connection and mechanism are required.

To elucidate the fitness of the mutants against the host's immune defenses, we evaluated the intracellular survival of the attenuated mutant strains in RAW 264.7 murine macrophages. In comparison to WT CO92, all the mutant strains evaluated exhibited significantly decreased intracellular survival, with the exception of Δ*ypo2720-2733* and Δ*ypo*2720-2733Δ*hcp3* (Figure 6C), suggesting that decreased fitness of these two mutants in host macrophages is not involved in their attenuation in animals.

The T6SSs are involved in the delivery of effector proteins to both prokaryotic and eukaryotic cells and, hence, can cause toxicity to the target cells (Russell et al., 2014). Out of the 10 T6SS deletion mutant strains tested, 7 strains exhibited decreased host cell cytotoxicity in comparison to WT CO92 (Figure 6A). For the Δ*ypo2720-2733* and Δ*ypo2720-2733*Δ*hcp3* mutant strains, which exhibited no defects in intracellular survival in murine macrophages, this decrease in cytotoxicity may be responsible for not only the attenuation observed for both mutants *in vivo*, but also subsequent protection from re-challenge with WT CO92 (Figure 4).

The Hcp protein is one of the most well characterized effectors of the T6SS (Filloux, 2013; Russell et al., 2014). In addition to its role as a structural component and secreted effector, in *A. dhakensi*s we have reported that Hcp is anti-phagocytic in nature (Suarez et al., 2010). As Hcp6 of *Y. pestis* shares significant homology (82%) with the Hcp protein of *A. dhakensis*, we evaluated whether Hcp6 plays a similar role in *Y. pestis*. In macrophages infected with Δ*vasK*, Δ*hcp6*, or Δ*vasK*Δ*hcp6* mutant strains, all exhibited similar and significantly higher rates of phagocytosis in comparison to WT CO92 (Figure 6B).

Indeed, in this same model of infection, the addition of rHcp6 (*Y. pestis*) or rHcp (*A. dhakensis*) decreased the percentage of phagocytosed bacteria for all three strains (Δ*vasK*, Δ*hcp6*, and Δ*vasK*Δ*hcp6*) reverting the phenotype observed back to that of WT CO92 (Figures 7A,B). These data indicated that Hcp6 of *Y. pestis* plays a similar role to that observed for Hcp in *A. dhakensis* and the rHcp/rHcp6 of these two pathogens can be interchangeably used (Figures 7A,B). We previously reported no defect in secretion of *hcp6* in the Δ*vasK* deletion mutant of *Y. pestis* when grown in culture, as observed by immunoblot analysis (Ponnusamy et al., 2015). However, increased phagocytosis of this deletion mutant and subsequent reversion to the WT phenotype upon addition of rHcp6 (Figure 7A) suggest a defect in either protein translocation or secretion in the host-pathogen co-culture model and warrants further investigation. For the other *hcp* homolog deletion mutants, only Δ*hcp4* exhibited any significant increase in phagocytosis in comparison to WT CO92 (Figure 6B). For the cluster deletion mutants, only Δ*ypo3588-3615* (Cluster G; Table 1), which includes *vasK*, exhibited any significant increase in phagocytosed bacteria (Figure 6B).

In summary, we have further characterized the role of four new virulence factors of *Y. pestis* through the generation of deletion mutants Δ*ypo0815*, Δ*ypo2884*, Δ*cyoABCDE*, and Δ*ypo1119-1120* (Table 2), which were identified through our STM screen. Additionally, we were able to further elucidate the role of the T6SS in regard to *Y. pestis* CO92 virulence through the generation of 12 T6SS loci, effector, and component deletion mutants and 2 combinatorial deletion mutants (Table 3).

**Table 2:** Summary of results for non-T6SS associated mutants in mice.

**Strain**	**% Animal survival in bubonic plague challenge**	**% Animal survival in pneumonic plague challenge**	**% Animal survival following rechallenge with WT CO92 (to gauge vaccine potential)**
Δ*cyoABCDE*	20	30	NT
Δ*ypo0815*	60	0	NT
Δ*ypo2884*	50	0	NT
Δ*lpp*Δ*cyoABCDE*	NT	90	55
Δ*lpp*Δ*ypo0815*	NT	50	20
Δ*lpp*Δ*ypo2884*	NT	60	42
Δ*ypo1119*	NT	0	NT
Δ*ypo1120*	NT	20	NT
Δ*intergenic:ypo1119-1120*	NT	100	NT
Δ*ypo1119-1120*	NT	100	0–20^*^
Δ*tolR*	NT	0	NT

* *LD_50_s of 50 and 100 were tested, resulting in range of survival of 0–20%; NT, not tested*.

**Table 3:** Summary of results for T6SS-associated mutant strains in mice and *in vitro* assays.

**Strain**	**% Animal survival in pneumonic plague challenge**	**% Animal survival following rechallenge with WT CO92 (to gauge vaccine potential)**	**Effect on cytotoxicity to macrophages**	**Effect on phagocytosis in macrophages**	**Effect on bacterial intracellular survival in macrophages**
Δ*ypo0966-0984*	42	NT	No effect	No effect	Decrease
Δ*ypo1458-1493*	14	NT	NT	NT	NT
Δ*ypo2720-2733*	38	NT	Decrease	No effect	No effect
Δ*ypo2927-2954*	0	NT	NT	NT	NT
Δ*ypo3588-3615*	30	NT	Decrease	Increase	Decrease
Δ*hcp3*	40	NT	Decrease	No effect	Decrease
Δ*hcp4*	42	NT	Decrease	Increase	Decrease
Δ*hcp5*	33	NT	No effect	No effect	Decrease
Δ*hcp6*	30	NT	Decrease	Increase	Decrease
Δ*ypo0873*	0	NT	NT	NT	NT
Δ*ypo1484*	20	NT	NT	NT	NT
Δ*ypo3615*	0	NT	NT	NT	NT
Δ*vasK*	20^a^	NT	No effect	Increase	Decrease
Δ*ypo2720-2733*Δ*hcp3*	60	100	Decrease	No effect	No effect
Δ*vasK*Δ*hcp6*	60	40	Decrease	Increase	Decrease

a *From previous study (Ponnusamy et al., 2015); NT, not tested*.

For the combinatorial deletion mutants Δ*lpp*Δ*cyoABCDE*, Δ*vasK*Δ*hcp6*, and Δ*ypo2720-2733*Δ*hcp3*, all provided statistically significant protection against subsequent re-challenge with WT CO92. Through *in vitro* studies, the attenuated T6SS mutant strains exhibited distinct phenotypes in terms of cytotoxic effects, resistance to phagocytosis by murine macrophages, and their intracellular survival in macrophages. These results indicated that the T6SS effectors and clusters have distinct roles in terms of *Y. pestis* virulence. Our future studies will focus on further delineating the role of these genes identified by our STM screening and those that are uncharacterized, as well as the T6SS in the pathogenesis of *Y. pestis* infection. These studies will aid in the continued development of live-attenuated vaccine candidates based on the combinatorial deletion of targeted *Y. pestis* virulence genes.

## Author contributions

JA, JS, TE, EF, DP, EK, and AC designed the experiments. JA, JS, TE, EF, DP, and EK designed and generated the mutant strains. JA, JS, TE, EF, DP, and MK performed the animal experiments. JA, JS, TE, EF, DP, EK, MK, and AC analyzed the data. JA, JS, and AC wrote and edited the manuscript. AC directed the project.

### Conflict of interest statement

Merck & Co., Inc. provided a monetary research support award to JA as a Maurice R. Hilleman Early-Stage Career Investigator in conjunction with the National Foundation for Infectious Diseases (NFID), but did not have any additional role in the study design, data collection, and analysis, decision to publish, or preparation of the manuscript. The other authors declare that the research was conducted in the absence of any commercial or financial relationships that could be construed as potential conflicts of interest.
