# Supplementary material for: Identification of New Virulence Factors and Vaccine Candidates for Yersinia pestis
Source: Front Cell Infect Microbiol. 2017 Oct 17;7:448. doi: 10.3389/fcimb.2017.00448 (PMC5650977; doi:10.3389/fcimb.2017.00448)
Supplement: Supplementary file 1 [file Table1.docx]

**
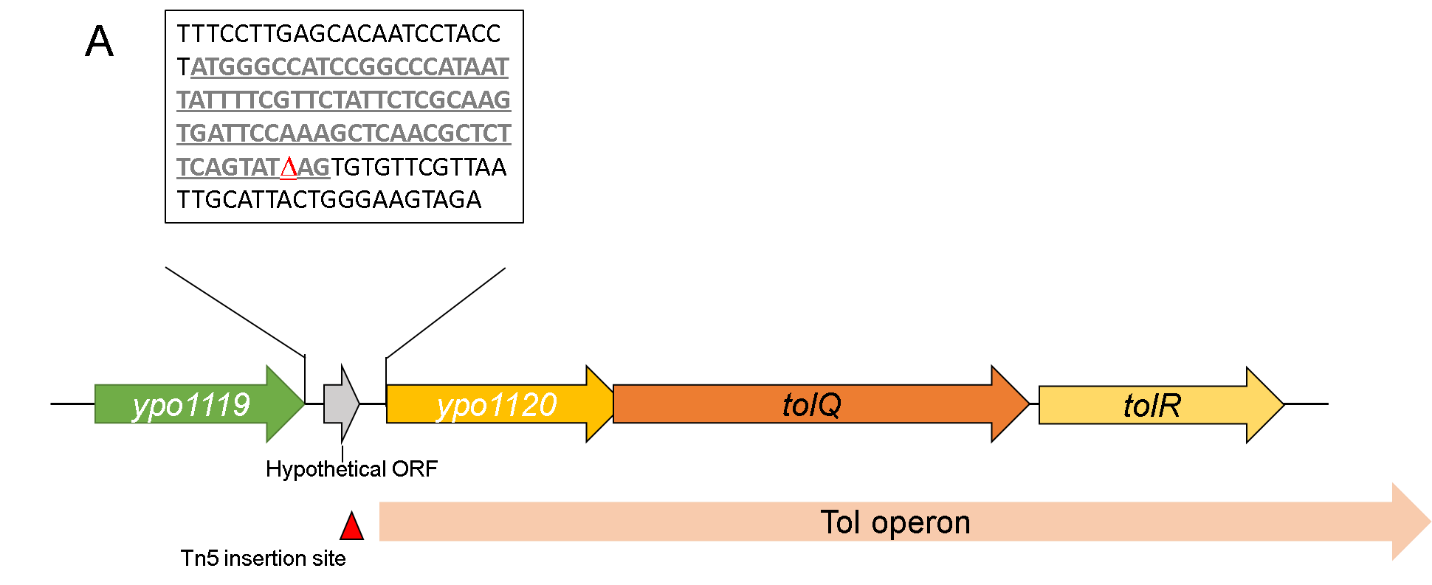

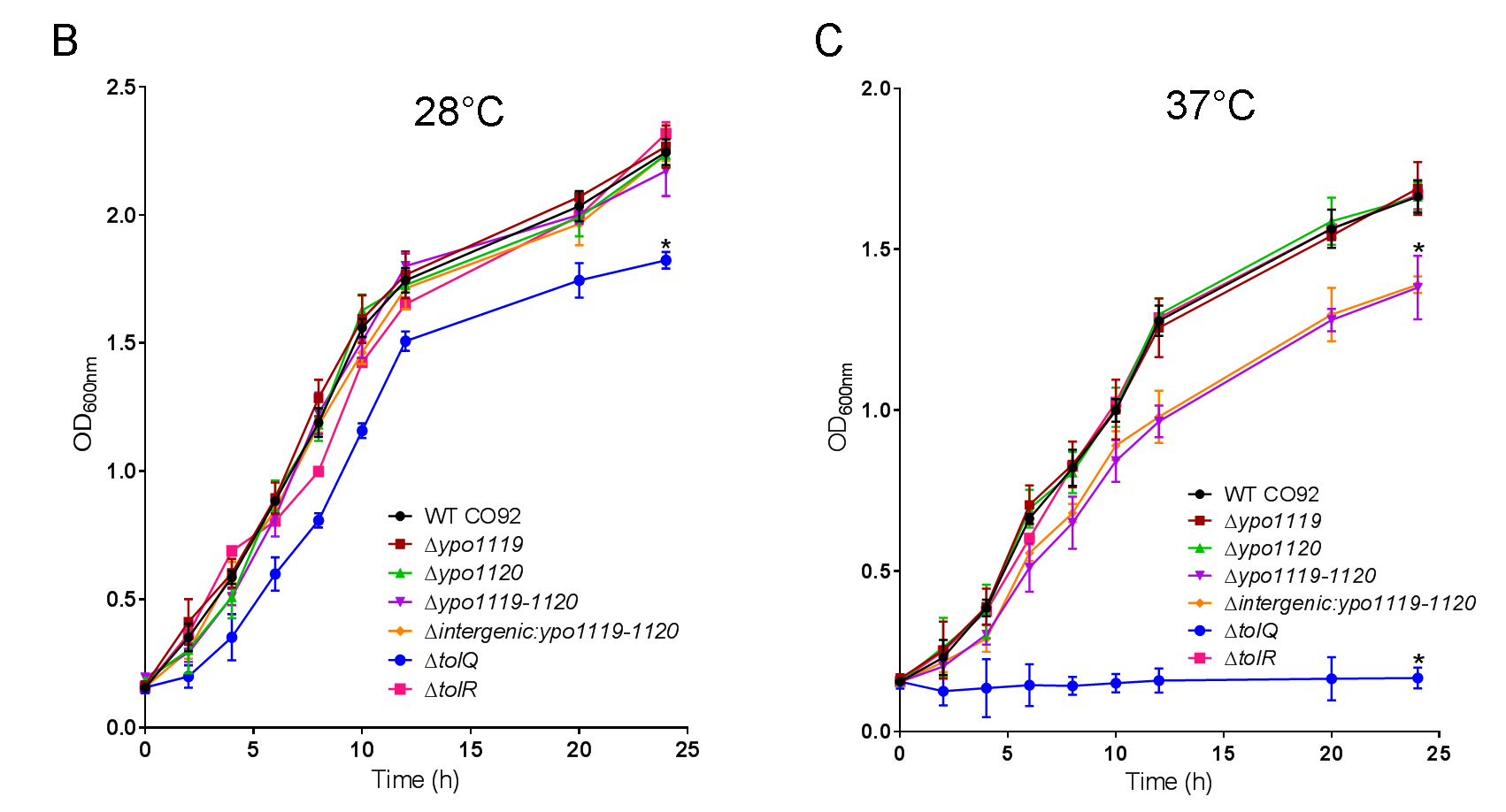
**

**Supplemental Figure 1: Schematic and growth of *ypo1119-1120* associated mutants.** (**A**) Schematic illustration of *ypo1119* and *ypo1120* with Tn*5* insertion site, hypothetical open reading frame (ORF) and the downstream genes of the YPO1120 operon, *tolQ* and *tolR*. (**B&C**). Growth of *ypo1119-1120* associated mutant strains in HIB at 28 (**B**) and 37°C (**C**) in comparison to WT CO92. Samples were taken at the indicated time points for OD_600_ measurement. Data shown are the mean values, while error bars represent standard deviations (SD) (2 replicates from 2 independent experiments; *n* = 4). Statistical analysis was performed using Student's *t* test. *, *P* ≤ 0.01, Δ = Tn5 insertion site.


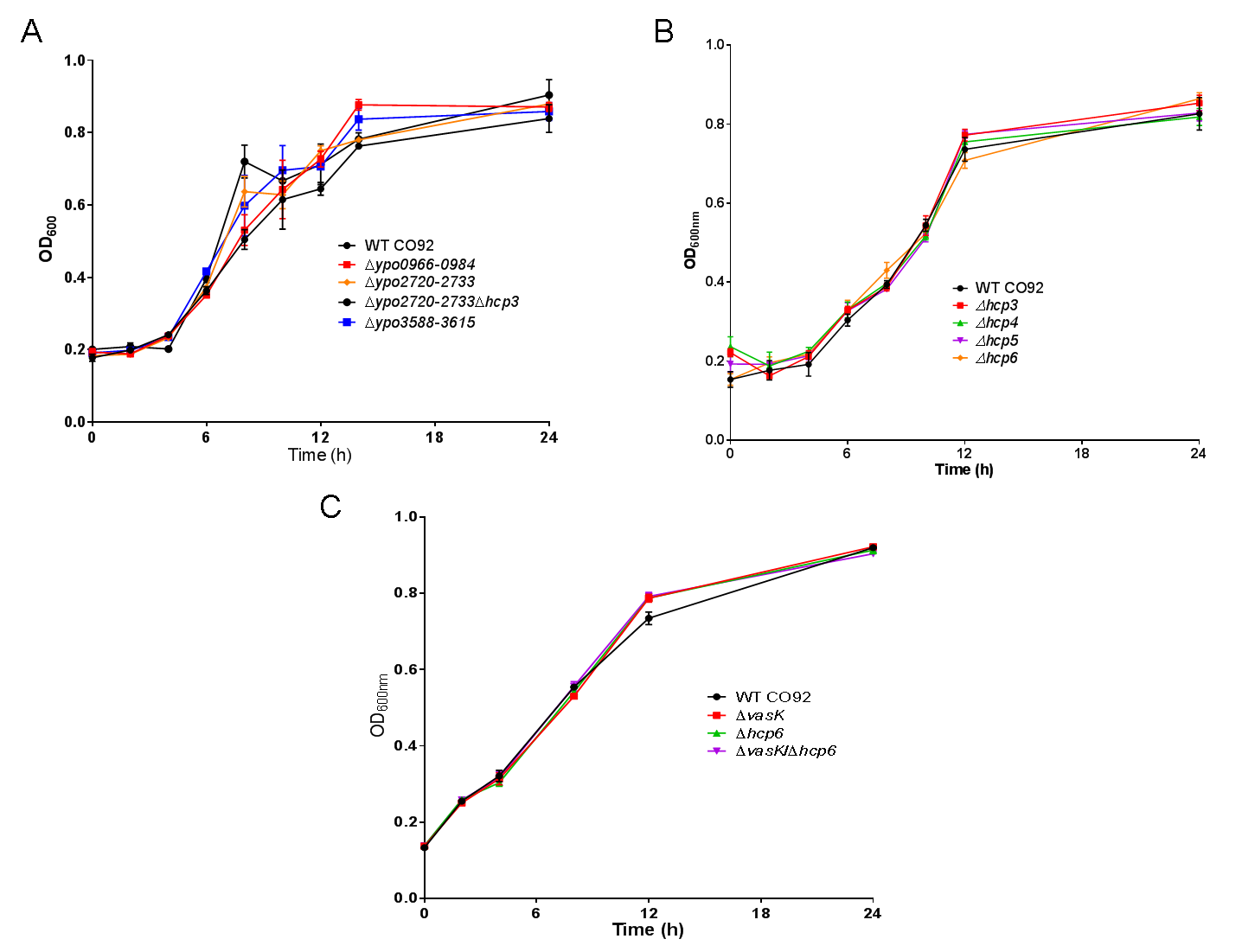


**Supplemental Figure 2:** **Growth of *Y. pestis* CO92 T6SS mutant strains** ***in vitro* 28°C***.* WT CO92 and the attenuated T6SS mutant strains were grown in HIB at 28° (**A**). Growth of the Δ*ypo0966-0984*, *Δypo2720-2733*, and Δ*ypo3588-3615* cluster deletion mutants and the double deletion mutant *Δypo2720-2733Δhcp3* in comparison to WT CO92. (**B**) Growth of *hcp* homolog deletion mutants Δ*hcp3,* Δ*hcp4,* Δ*hcp5,* and Δ*hcp6* in comparison to WT CO92*.* (**C**) Growth of Δ*vasK,* Δ*hcp6,* and Δ*vaskhcp6* in comparison to WT CO92. Samples were taken at the indicated time points for OD_600_ measurements. Data shown are the mean values, while error bars represent standard deviations (SD) (2 replicates from 2 independent experiments; *n* = 4). Statistical analysis was performed using Student's *t* test, with no mutant strains exhibiting any significant differences in growth in comparison to WT CO92.

**
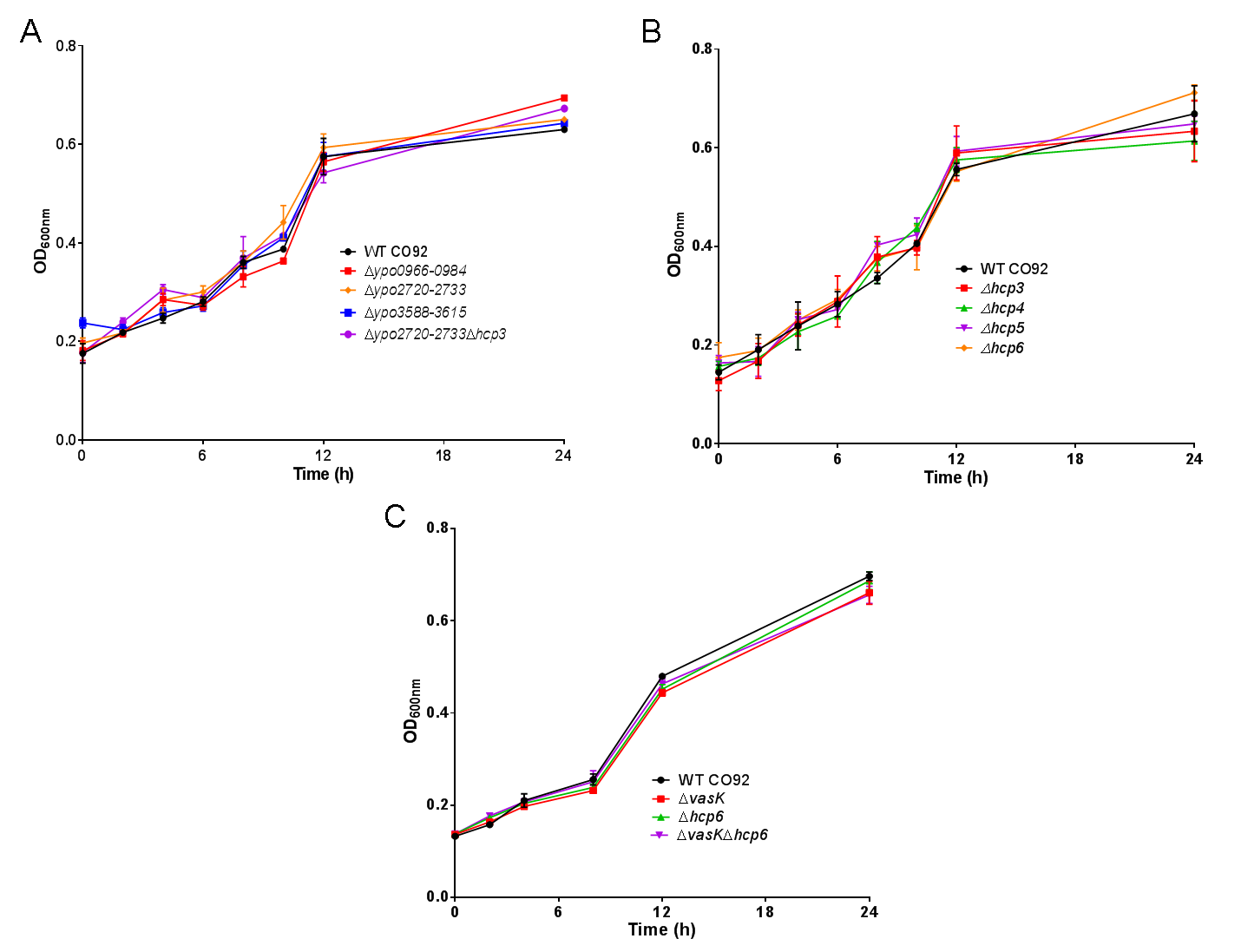
Supplemental Figure 3:** **Growth of *Y. pestis* CO92 T6SS mutant strains** ***in vitro* at 37°C***.* WT CO92 and the attenuated T6SS mutant strains were grown in HIB at 37°C. (**A**) Growth of the Δ*ypo0966-0984*, *Δypo2720-2733*, and Δ*ypo3588-3615* cluster deletion mutants and the double deletion mutant *Δypo2720-2733Δhcp3* in comparison to WT CO92. (**B**) Growth of *hcp* homolog deletion mutants Δ*hcp3,* Δ*hcp4,* Δ*hcp5,* and Δ*hcp6* in comparison to WT CO92. (**C**) Growth of Δ*vasK,* Δ*hcp6,* and Δ*vaskhcp6* in comparison to WT CO92. Samples were taken at the indicated time points for OD_600_ measurements. Data shown are the mean values, while error bars represent standard deviations (SD) (2 replicates from 2 independent experiments; *n* = 4). Statistical analysis was performed using Student's *t* test, with no mutant strains exhibiting any significant differences in growth in comparison to WT CO92.

**Table S1: Bacterial strains and plasmids used in this study**

| Strain or plasmid | Description | Reference or source |
| --- | --- | --- |
| *Y. pestis* CO92 strains |  |  |
| WT CO92 | Virulent *Y. pestis* biovar Orientalis strain isolated in 1992 from a fatal human pneumonic plague case and naturally resistant to polymyxin B | CDC |
| WT CO92:pBR322 | WT *Y. pestis* CO92 transformed with tetracycline sensitive (Tc^s^) variant pBR322 | van Lier et al., 2014 |
| WT CO92:pKD46 | WT *Y. pestis* CO92 transformed with plasmid pKD46 encoding phage λ Red recombinase | Ponnusamy et al., 2015 |
| Δ*vasK* | vasK (ypo3603) deletion mutant of *Y. pestis* CO92 | Ponnusamy et al., 2015 |
| Δ*lpp* | lpp deletion mutant of Y. pestis CO92 | Sha et al., 2008 |
| Δ*ypo0815* | ypo0815 deletion mutant of Y. pestis CO92 | This study |
| Δ*lpp*Δ*ypo0815* | lpp and ypo0815 double deletion mutant of Y. pestis CO92 | This study |
| Δ*ypo2884* | ypo2884 deletion mutant of Y. pestis CO92 | This study |
| Δ*lpp*Δ*ypo2884* | lpp and ypo2884 double deletion mutant of Y. pestis CO92 | This study |
| Δ*cyoABCDE* | cyoABCDE deletion mutant of Y. pestis CO92 | This study |
| Δ*lpp*Δ*cyoABCDE* | lpp and cyoABCDE double deletion mutant of Y. pestis CO92 | This study |
| Δ*intergenic:ypo1119-1120* | intergenic region between ypo1119-1120 deletion mutant of Y. pestis CO92 | This study |
| Δ*ypo1119-1120* | ypo1119-1120 whole locus deletion mutant of Y. pestis CO92 | This study |
| Δ*ypo1119* | ypo1119 deletion mutant of Y. pestis CO92 | This study |
| Δ*ypo1120* | ypo1120 deletion mutant of Y. pestis CO92 | This study |
| Δ*tolQ* | tolQ deletion mutant of Y. pestis CO92 | This study |
| Δ*tolR* | tolR deletion mutant of Y. pestis CO92 | This study |
| Δ*ypo0499-0516* | Partial T6SS cluster A deletion mutant of *Y. pestis* CO92 | Robinson et al., 2009 |
| Δ*ypo0966-0984* | T6SS cluster B deletion mutant of *Y. pestis* CO92 | This study |
| Δ*ypo1458-1493* | T6SS cluster C deletion mutant of *Y. pestis* CO92 | This study |
| Δ*ypo2720-2733* | Partial T6SS cluster E deletion mutant of *Y. pestis* CO92 | This study |
| Δ*ypo2927-2954* | T6SS cluster F deletion mutant of *Y. pestis* CO92 | This study |
| Δ*ypo3588-3615* | T6SS cluster G deletion mutant of *Y. pestis* CO92 | This study |
| Δ*hcp3* | *ypo2793* deletion mutant of *Y. pestis* CO92 | This study |
| Δ*hcp3*:pBR322-*hcp3* | Δhcp3 complemented with pBR322-hcp3 (Tc^s^, Ap^r^) | This study |
| Δ*hcp4* | *ypo2868* deletion mutant of *Y. pestis* CO92 | This study |
| Δ*hcp4*:pBR322-*hcp4* | Δhcp4 complemented with pBR322-hcp4 (Tc^s^, Ap^r^) | This study |
| Δ*hcp5* | *ypo2962* deletion mutant of *Y. pestis* CO92 | This study |
| Δ*hcp5*:pBR322-*hcp5* | Δhcp5 complemented with pBR322-hcp5 (Tc^s^, Ap^r^) | This study |
| Δ *hcp6* | *ypo3708* deletion mutant of *Y. pestis* CO92 | This study |
| Δ*hcp6*:pBR322-*hcp6* | Δhcp6 complemented with pBR322-hcp6 (Tc^s^, Ap^r^) | This study |
| Δ*ypo0873* | *ypo0873* deletion mutant of *Y. pestis* CO92 | This study |
| Δ*ypo1484* | *ypo1484* deletion mutant of *Y. pestis* CO92 | This study |
| Δ*ypo3615* | *ypo3615* deletion mutant of *Y. pestis* CO92 | This study |
| Δ*vasK* Δ*hcp6* | *vasK* (*ypo3603*) and hcp6 (*ypo3708*) double deletion mutant of *Y. pestis* CO92 | This study |
| Δ*ypo2720-2733* Δ*hcp3* | Partial T6SS cluster E and hcp3 (*ypo2793*) double deletion mutant of *Y. pestis* CO92 | This study |
| Plasmids |  |  |
| pKD46 | Plasmid for phage λ Red recombinase under arabinose-inducible promoter (Ap^r^) | Datsenko and Wanner, 2000 |
| pKD13 | Template plasmid for PCR amplification of the Km^r^ gene cassette flanked by FRT sites | Datsenko and Wanner, 2000 |
| pEF01 | Plasmid for FLP enzyme under constitutively expressed lac promoter (Ap^r^) | Fitts et al., 2016 |
| pBR322 | A tetracycline sensitive (Tc^s^) variant of pBR322 | Galindo et al., 2010 |
| pRB322-*hcp3* | Plasmid containing the hcp3 (*ypo2793*) coding region and its putative promoter inserted in the Tc^r^ cassette of vector pBR322 (Tc^s^, Ap^r^) | This study |
| pRB322-*hcp4* | Plasmid containing the hcp4 (*ypo2868*) coding region and its putative promoter inserted in the Tc^r^ cassette of vector pBR322 (Tc^s^, Ap^r^) | This study |
| pRB322-*hcp5* | Plasmid containing the hcp5 (*ypo2962*) coding region and its putative promoter inserted in the Tc^r^ cassette of vector pBR322 (Tc^s^, Ap^r^) | This study |
| pRB322-*hcp6* | Plasmid containing the hcp6 (*ypo3708*) coding region and its putative promoter inserted in the Tc^r^ cassette of vector pBR322 (Tc^s^, Ap^r^) | This study |
| pET-30a | pBR322-derived expression vector with T7 lac promotor, up- and down- stream His tags; Km^r^. | Novagen |
| pET-30a-*hcp6* | pET-30a with the coding region of *hcp6* used to produce the His-tagged recombinant Hcp6 (rHcp6) (Km^r^) | This study |

^a^CDC, Centers for Disease Control and Prevention; Tc^r^, tetracycline resistance; Tc^s^, tetracycline susceptibility; Ap^r^, ampicillin resistance; FLP, flippase; FRT, flippase recognition target.

**Table S2: Primers used in this study**^a^

| **Primer or primer pair** | | **Primer sequences (5’ – 3’; forward, reverse)** | **Purpose** |
| --- | --- | --- | --- |
| 1 | Km*ypo0815* | AGTATTAGGAAAGATATAAATAATTTTTATACACTGTTGGACTCAGAGCTGTGTAGGCTGGAGCTGCTTC (FRT sequence),  CATCAATATCACCTGTTATCTTGGTTCCACTTCTGCTGATGGCAACATATATTCCGGGGATCCGTCGACC (FRT sequence) | Construction of a DNA fragment with Km^r^ gene cassette and FRT sequence for the *ypo0815* mutation |
| 2 | *ypo0815*V | TACGATTGTTAATAAAGTCCCCTTC,  AACTCTAATCCTTTGAATTATTTCA | PCR verification of ypo0815 deletion |
| 3 | Km*ypo2884* | TATTTTTAAAATAATGGATTATTAATAATAGTTTAAAAGGTATATATATTGTGTAGGCTGGAGCTGCTTC (FRT sequence),  TAATAATGTTTAATTTATCTTTTTTTCCAATCAGTGAGTACCCAATATCCATTCCGGGGATCCGTCGACC (FRT sequence) | Construction of a DNA fragment with Km^r^ gene cassette and FRT sequence for the *ypo*2884 mutation |
| 4 | *ypo2884*V | CGATCCATGGATCTCGCCTTGTCTC,  TAACCGGACTCGGAACCTGCTCTGA | PCR verification of ypo2884 deletion |
| 5 | Km*cyoABCDE* | CCCGCACACATGAAACGACAATAGAGCCATTTTGTAATTGAGGTCGTTAGGTGTAGGCTGGAGCTGCTTC (FRT sequence),  GATTCCTGCCAGCCACTTGATTTATTTCACTCTCATCATTTTCGCTAAGAATTCCGGGGATCCGTCGACC (FRT sequence) | Construction of a DNA fragment with Km^r^ gene cassette and FRT sequence for the *cyoABCDE* mutation |
| 6 | *cyoABCDE*V | ACATATTATTCACACGAAATAAGTT,  TTTTGTAGGCATCTGATTATAGGTA | PCR verification of cyoABCDE deletion |
| 7 | Km*ypo1119-1120* | ACTTCTGGCCTGCTCCTTTGGTATCATCACTGCTCTGGCATTGGAGCAGAGTGTAGGCTGGAGCTGCTTC (FRT sequence),  AACGAATAGGCTTGCCTGCAGGAATAAATCCAGAATGTTCATGTCAGCCAATTCCGGGGATCCGTCGACC (FRT sequence) | Construction of a DNA fragment with Km^r^ gene cassette and FRT sequence for the *ypo1119-1120* mutation |
| 8 | *ypo1119-1120*V | TCCCGTTCGTTATGCCATCAAGCAC,  GTGCATGGCTGTTTGCACGATGTAG | PCR verification of ypo1119-1120 deletion |
| 9 | Km*intergenic:ypo1119-1120* | TTCCTGCAATTGTGATCTTGGCTACAGGATTATTCTACTTTTTCGCCTAAGTGTAGGCTGGAGCTGCTTC (FRT sequence),  GTGAGTAATACGTTATTTCGATGGCCGGTACGAGTCTACTTTGAAGATACATTCCGGGGATCCGTCGACC (FRT sequence) | Construction of a DNA fragment with Km^r^ gene cassette and FRT sequence for the *intergenic:ypo1119-1120* mutation |
| 10 | *intergenic:ypo1119-1120*V | TCCCGTTCGTTATGCCATCAAGCAC,  GTGCATGGCTGTTTGCACGATGTAG | PCR verification of *intergenic:*ypo1119-1120 deletion |
| 11 | Km*ypo1119* | ACTTCTGGCCTGCTCCTTTGGTATCATCACTGCTCTGGCATTGGAGCAGAGTGTAGGCTGGAGCTGCTTC (FRT sequence),  AAATAATTATGGGCCGGATGGCCCATAGGTAGGATTGTGCTCAAGGAAAAATTCCGGGGATCCGTCGACC (FRT sequence) | Construction of a DNA fragment with Km^r^ gene cassette and FRT sequence for the *ypo1119* mutation |
| 12 | *ypo1119*V | TCCCGTTCGTTATGCCATCAAGCAC,  AAGCAACATGCTCACTGAGCAATTG | PCR verification of ypo1119 deletion |
| 13 | Km*ypo1120* | CAACGCTCTTCAGTATAGTGTGTTCGTTAATTGCATTACTGGGAAGTAGAGTGTAGG (FRT sequence),  AACGAATAGGCTTGCCTGCAGGAATAAATCCAGAATGTTCATGTCAGCCAATTCCGG (FRT sequence) | Construction of a DNA fragment with Km^r^ gene cassette and FRT sequence for the *ypo1120* mutation |
| 14 | *ypo1120*V | CACCAGCTCACTGGAAGTATGGCAG,  GTGCATGGCTGTTTGCACGATGTAG | PCR verification of ypo1120 deletion |
| 15 | Km*tolQ* | AAGAGCGCTTCCTAAGTCTATTGTCGCGGAGTTTAAGTAGTGGCTGACATGTGTAGG (FRT sequence),  GCCATATAACCCCCTACTTATTTGCTTTCGGTGCTGGCGAAAGCCTGGCGATTCCGG (FRT sequence) | Construction of a DNA fragment with Km^r^ gene cassette and FRT sequence for the *tolQ* mutation |
| 16 | *tolQ*V | GGCGCGAACTGAAATGTTGCGCCAA,  TTGAGCTTCTGCCACCACTTGCTCT | PCR verification of tolQ deletion |
| 17 | Km*tolR* | GGCTTTCGCCAGCACCGAAAGCAAATAAGTAGGGGGTTATATGGCGCGAGGTGTAGG (FRT sequence),  TGAGCTAACGATTAGCCCAACCAATATCAACGCAAATTTATATCGGTTGGATTCCGG (FRT sequence) | Construction of a DNA fragment with Km^r^ gene cassette and FRT sequence for the *tolR* mutation |
| 18 | *tolR*V | CAACGGATGGAGCTGTTGCCTTCAG,  CTGCACCTACGATGGCGCCAATTTC | PCR verification of tolR deletion |
| 19 | Km*ypo0966-0984* | CTGAAGACCCCATTAAACGTTGGCTATTCTCTTAACAGGACGTTAAAAATGTGTAGGCTGGAGCTGCTTC (FRT sequence),  TGTAATTAACGCAAAACAGGCAATCGGGCTTGGTGTTGAACTGGAAATTAATTCCGGGGATCCGTCGACC (FRT sequence) | Construction of a DNA fragment with Km^r^ gene cassette and FRT sequence for the *ypo*0966-0984 mutation |
| 20 | *ypo0966-0984*V | ACTCCCCCTTCCTTGCAATTAATCACTTCA,  ATGATAGGGACAGGCTACAAATGCT | PCR verification of ypo0966-0984 deletion |
| 21 | Km*ypo1458-1493* | TGGGCTCCTTATTCTTGAGCGTATCGATAACCACTTACGGCGTTATCGCTGTGTAGGCTGGAGCTGCTTC (FRT sequence),  TAATTGCCTGTTTTTGATATCTTCACTCCAACAACGGAGACAGGCAAATTATTCCGGGGATCCGTCGACC (FRT sequence) | Construction of a DNA fragment with Km^r^ gene cassette and FRT sequence for the *ypo*1458-1493 mutation |
| 22 | *ypo1458-1493*V | GAATAGGGGTCTACTCTTATGGTTC,  AATTTACAGGCAATGGAAGCCATAAAGCTG | PCR verification of ypo1458-1493 deletion |
| 23 | Km*ypo2720-2733* | AAGTGAAATCAGGCTGGGCAGATGACGAACGTGCATTGCGCAGCTTAGGCTATACCGACGATTTGAAATAAGGCTTGCCGGTGTAGGCTGGAGCTGCTTC (FRT sequence),  ATTACACTGCGACGGCGGTTTTCAGTTGCTGAAGATTATTGATGATATAACCTCCCCCTAATCGCTCTCACTAATTATTTATTCCGGGGATCCGTCGACC (FRT sequence) | Construction of a DNA fragment with Km^r^ gene cassette and FRT sequence for the *ypo*2720-2733 mutation |
| 24 | ypo2720-2733V | GGCGAAGAACTGCCTTATTCGGTGACGGTT,  AGTCCATCGACGTTAACATTTCATGATGTT | PCR verification of ypo2720-2733 deletion |
| 25 | Km*ypo2927-2954* | GTAGGTTATGTAAAGTGGCAAGCGATGAGATATGTCATTAACTGCATGATTTAAAAAGATTAAAAAGGTAAGTTACTGAAGTGTAGGCTGCAGCTGCTTC (FRT sequence),  GCGATGTTTAACATATTCTTCCAATACGACCACGTCATGCTAAGTCGGGTTATTTAGCATGACGTGATATCGCTCAAAAAATTCCGGGGATCCGTCGACC (FRT sequence) | Construction of a DNA fragment with Km^r^ gene cassette and FRT sequence for the *ypo*2927-2954 mutation |
| 26 | *ypo2927-2954*V | CCGTAGCGCGGCGATCTCTTCCAGTACCGC,  GGAATGAGCTGTAGATAGGCCCGTTACTGC | PCR verification of ypo2927-2954 deletion |
| 27 | Km*ypo3588-3615* | AACAGAACCAAGTTAAGCATTACGACTCATTAAAATAAGGGTAGCCCGCGACCACGGGTAAGCACAGAGGAGAGCAAAAAGTGTAGGCTGGAGCTGCTTC (FRT sequence),  TAGCATACCTATTTCATATGCAGTTTCAGGAGGGATATTCTCAGTACCATAAATAGCTATAGCCGTACACATAGTTTCACATTCCGGGGATCCGTCGACC (FRT sequence) | Construction of a DNA fragment with Km^r^ gene cassette and FRT sequence for the *ypo2927-2954* mutation |
| 28 | *ypo3588-3615*V | AAAAAGACGATCTTTACTCCTTGCAGAGAA,  AACCACCAAAATCACTTGTTGAGAACCCAA | PCR verification of ypo3588-3615 deletion |
| 29 | Km*ypo2793* | GATTATCGGTTTATACTCAGGCTTGAAGTGCCGCCAATTTTGGAACGACAGCACTAACCTCTATTACATAAGGAATTGTCGTGTAGGCTGGAGCTGCTTC (FRT sequence),  AAGCTATGCTCAAGATCATCTTGGATGTCCCCTAATGAATACGTAAAAGATATAAAGCTATGTCCATAGTTAACCGCCTCATTCCGGGGATCCGTCGACC (FRT sequence) | Construction of a DNA fragment with Km^r^ gene cassette and FRT sequence for the *ypo2793* mutation |
| 30 | *ypo2793*V | TCTTTTTTAATAAGAATGGGGTGACATGAT,  TGATGTTATTATCTTCTCATGGAAAGTTGG | PCR verification of ypo2793 deletion |
| 31 | *ypo2793*C | GTAAGCTTAACGTCAACGACCAGTAAGTG (HindIII),  GCGGATCCGCTATGTCCATAGTTAACCG (BamHI) | Cloning of *ypo*2793 and its promoter region in plasmid pBR322 |
| 32 | Km*ypo2868* | GTAAGAGACTTATCACTGTGTAAATAACAACTTTAAATATAAGGAATTGTGTGTAGGCTGGAGCTGCTTC (FRT sequence),  TTATGGTTTTTAACGATATAGGGTATATGCCTATAGCCGTCACTTAAAAAATTCCGGGGATCCGTCGACC (FRT sequence) | Construction of a DNA fragment with Km^r^ gene cassette and FRT sequence for the *2868* mutation |
| 33 | *ypo2868*V | ATTCTCTAGCTCCATAGCAATATGC,  TGTCGAGGGTATTACGTTGATACGG | PCR verification of ypo2868 deletion |
| 34 | *ypo2868*C | GTAAGCTTTGTTAGCATACGAG (HindIII),  CAGGATCCTATATGCCTATAGCCGTCAC (BamHI) | Cloning of *ypo*2868 and its promoter region in plasmid pBR322 |
| 35 | Km*ypo2962* | AAGGGCAACTAACCCCCTGCATCTTGAAGGCGACGGGTATATAAGGACATGTGTAGGCTGGAGCTGCTTC (FRT sequence),  CGTATGTACACGTAAATAATAAGGGATGGTGTAGGCCATCCCCGAATAAAATTCCGGGGATCCGTCGACC (FRT sequence) | Construction of a DNA fragment with Km^r^ gene cassette and FRT sequence for the *ypo2962* mutation |
| 36 | *ypo2962*V | TCCCGCCAGCGATAACGCTCCCGTCAGATA,  GTTCAATGGGTCTGTTGGCTGAGTTTTTTG | PCR verification of ypo2962 deletion |
| 37 | *ypo2962*C | ATAAGCTTCCGTCAGATAATAGTAGCGG (HindIII),  ATGGATCCTTAATACACGCGATCATCCCATATGC (BamHI) | Cloning of ypo2962 and its promoter region in plasmid pBR322 |
| 38 | Km*ypo3708* | AAGTGACTAACCTTTGATCAAAATCAAATAAACTATCAAGGATATTAAAAGTGTAGGCTGGAGCTGCTTC (FRT sequence),  CGCTTCGATCGGCGCACGCCAGTCATCAGCACCAGAGGTGCCCGCGGTGGATTCCGGGGATCCGTCGACC (FRT sequence) | Construction of a DNA fragment with Km^r^ gene cassette and FRT sequence for the *hcp6* mutation |
| 39 | *ypo3708*V | CGAAACTTGGCACTGATAAAAAAGC,  TAATATATCCCTGGCGGCGACACAT | PCR verification of hcp6 deletion |
| 40 | *ypo3708*C | CGGGATCCTTTTGTAACATTTGCGAATTAA (BamHI),  ACGCGTCGACTTACGCTTCGATCGGCGCAC (SaII) | Cloning of hcp6 and its promoter region in plasmid pBR322 |
| 41 | *ypo3708*R | TCATAGATCTAATGCCAACTCCAGCTTATATCTC (BglII),  GGTCCTCGAGTTACGCTTCGATCGGCGCACG (XhoI) | Cloning of hcp6 in plasmid pET30 vector |
| 42 | Km*ypo0873* | CATCATTATCCCTTACCTTTTTGGGCATATTGCCAAATTGATATTGGCAAACGGATAAATAAGGGATGATGTGTAGGCTGGAGCTGCTTC (FRT sequence),  GGGCAAATTTCATAATCCCCAAGATTATCTATTGTTTTTGATCCGCAGCACTGACATGAATATTCATCACATTCCGGGGATCCGTCGACC (FRT sequence) | Construction of a DNA fragment with Km^r^ gene cassette and FRT sequence for the *ypo0873* mutation |
| 43 | *ypo0873*V | TCTCCTAACCCCTACCAATCCCTTA,  AAAGCCACTGTTGCTGATAAACCGT | PCR verification of ypo0873 deletion |
| 44 | Km*ypo1484* | GGCTAATCGCCCTGCACCCGGCCCGTGCGGCCGTATTATGCGGATAACAGGTGTAGGCTGGAGCTGCTTC (FRT sequence),  TCCTAATACGATAAAAATCGTAACAACCATTATTATTCTTTTCATTACTTTTCCGGGGATCCGTCGACCTATTCCGGGGATCCGTCGACC (FRT sequence) | Construction of a DNA fragment with Km^r^ gene cassette and FRT sequence for the *ypo1484* mutation |
| 45 | *ypo1484*V | AAGTAATGAAAAGAATAATAATGGTTGTTACGAT,  CTTCTCTGTTATCCGCATAATACGGC | PCR verification of ypo1484 deletion |
| 46 | Km*ypo3615* | TCATAATTTACGGTCGTTTCGGATAGAGCCACGGCATCAGGAGGATGTTTGTGTAGGCTGGAGCTGCTTC (FRT sequence),  TAGCATACCTATTTCATATGCAGTTTCAGGAGGGATATTCTCAGTACCATAAATAGCTATAGCCGTACACATAGTTTCACATTCCGGGGATCCGTCGACC (FRT sequence) | Construction of a DNA fragment with Km^r^ gene cassette and FRT sequence for the *ypo3615* mutation |
| 47 | *ypo3615*V | AGGCTCTCTGAGCCGGCAATTAAATCACTA,  AACCACCAAAATCACTTGTTGAGAACCCAA | PCR verification of ypo3615 deletion |

^a^ Underlining indicates the restriction enzyme sites or FRT sequence sites in the primers, with the restriction enzymes or FRT sequence indicated in parentheses.
